# Maternal provisions in type 1 diabetes: Evidence for both protective & pathogenic potential

**DOI:** 10.3389/fimmu.2023.1146082

**Published:** 2023-03-22

**Authors:** Erin Strachan, Xavier Clemente-Casares, Sue Tsai

**Affiliations:** Department of Medical Microbiology & Immunology, University of Alberta, Edmonton, AB, Canada

**Keywords:** autoimmunity, immunoglobulin, autoantibody, microbiome, dysbiosis, tolerance

## Abstract

Maternal influences on the immune health and development of an infant begin *in utero* and continue well into the postnatal period, shaping and educating the child’s maturing immune system. Two maternal provisions include early microbial colonizers to initiate microbiota establishment and the transfer of antibodies from mother to baby. Maternal antibodies are a result of a lifetime of antigenic experience, reflecting the infection history, health and environmental exposure of the mother. These same factors are strong influencers of the microbiota, inexorably linking the two. Together, these provisions help to educate the developing neonatal immune system and shape lymphocyte repertoires, establishing a role for external environmental influences even before birth. In the context of autoimmunity, the transfer of maternal autoantibodies has the potential to be harmful for the child, sometimes targeting tissues and cells with devastating consequences. Curiously, this does not seem to apply to maternal autoantibody transfer in type 1 diabetes (T1D). Moreover, despite the rising prevalence of the disease, little research has been conducted on the effects of maternal dysbiosis or antibody transfer from an affected mother to her offspring and thus their relevance to disease development in the offspring remains unclear. This review seeks to provide a thorough evaluation of the role of maternal microorganisms and antibodies within the context of T1D, exploring both their pathogenic and protective potential. Although a definitive understanding of their significance in infant T1D development remains elusive at present, we endeavor to present what has been learned with the goal of spurring further interest in this important and intriguing question.

## Graphical Abstract

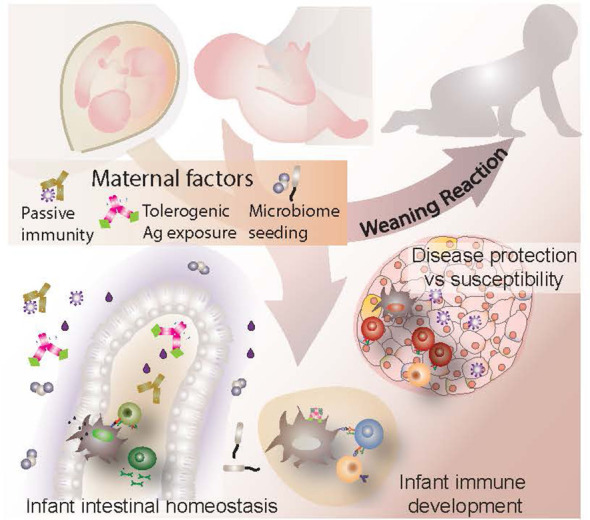

## 1 Introduction

Immunity is a balancing act that relies on accurate assessment and precise management of contrasting, often opposing factors. The effectiveness of our immune system is contingent on its ability to assess a threat correctly, respond appropriately, and resolve the threat without undue harm to the host. Thus, poor immune regulation leaves an individual vulnerable to self-mediated destruction. During development, T and B lymphocytes are subjected first to the process of central tolerance followed by tight regulation *via* various mechanisms of peripheral tolerance. Errors and malfunctions in tolerance education and regulation can give rise to autoimmune diseases, as is the case in Type 1 diabetes (T1D). Research over the last decades has expanded our understanding of the various events involved in onset and pathogenesis of T1D. Furthermore, it has illuminated the numerous factors contributing to disease, among them genetic, environmental, dietary and microbiome contributions, as well as early life events and conditions. In spite of its importance, there is a paucity of studies that elucidate maternal-derived mechanisms of T1D resistance vs pathogenesis. One could argue that genetic, dietary, lifestyle, environmental and microbiome contributors are all maternally rooted influences when viewed from a neonatal perspective. Of particular note, T1D studies worldwide have consistently reported a bias in disease prevalence amongst children of a parent with diabetes, with increased risk attributed to those with an affected father (1–11). This observation suggests that on top of genetic predisposition, an affected mother may bestow a protective phenotype upon children, an intriguing possibility that raises many questions about the role of maternal factors in autoimmune diseases. Furthermore, given the numerous maternal influences on neonatal immune development, coupled with the significance of the gestational and neonatal influences on lifelong immune function and resilience, the effects of maternal factors on the development of T1D cannot be over-emphasized.

The intestinal microbiota is a facet that has been widely investigated for both its role and response to T1D onset and pathogenesis. Human studies, as well as animal models, have consistently demonstrated changes in microbiome diversity, composition and activity in affected individuals both prior to and after diagnosis (12). Indeed, intestinal dysbiosis and its disease modifying consequences have been well explored and thoroughly reviewed by others (12–15) in so far as it applies to affected individuals, but relatively little work has been invested into how T1D associated dysbiosis in pregnant and breastfeeding mothers affects the infant, in terms of both microbiome establishment and immune development. Similarly, questions pertaining to the significance of T1D associated autoantibodies (AA) have been largely restricted to their involvement in disease progression in patients (16), while only a few have considered their role in perpetuating or curbing disease in the infant. Maternally acquired AA are a key element in some autoimmune diseases and could conceivably play a role in T1D. Moreover, maternally acquired antibodies participate in shaping the infant immune system and microbiome during the neonatal period. Thus, we propose that maternal provision of immunoglobulin, microorganisms and other immunomodulatory factors are important interdependent influencers of infant immune development and establishment of intestinal homeostasis. This review seeks to explore the various mechanisms by which these influences operate, with a specific focus on their significance in T1D (see graphical abstract).

## 2 The neonatal immune environment

The immune environment in a newborn is distinctly different from that of an older child or adult. Although all components of the innate and adaptive systems are present by the third trimester of gestation, many are present in lower concentrations or exhibit weak responses to stimuli, diminished chemotaxis, impaired cytotoxicity, or reduced expression of activation receptors (17). During this phase of development, B and T lymphocytes have limited receptor repertoires, and when challenged with foreign antigen, display a strong propensity towards a regulatory phenotype. In general, the neonatal immune environment can be characterized as anti-inflammatory, highly tolerogenic and amazingly malleable to external influences and manipulation (17).

Due to the largely sterile environment *in utero*, antigenic stimulation prior to birth is dominated by maternal alloantigens and autoantigens. Upon delivery, the neonate is bombarded by antigenic challenge primarily through respiratory and gastrointestinal avenues (18, 19). The challenge of this particular period of time is the need to balance an emerging functional immune system with the exponential growth and colonization of the gut microbiota, while at the same time providing sufficient immune protection for the infant. Furthermore, both the innate and adaptive arms of the immune system require maturation, education and expansion. This is a tall order, but one that is amply met *via* both maternal and endogenous mechanisms. Research into various avenues of immunity and disease have established that within childhood there exists a critical window of time, one that is vital to the successful development and education of one’s immune system. Immunological events that occur, or fail to occur, during this timeframe have the ability to shape one’s health in a lifelong fashion.

### 2.1 The weaning reaction

Insights into the neonatal period have been largely ascertained through animal studies in recent years. In mice, this window of time encompasses postpartum weeks 1-3 and the immunological events that occur have been termed the ‘weaning reaction’ (20). The weaning reaction occurs in the gastrointestinal (GI) tract, largely in the distal ileum and colon (20–22). From birth to approximately 10 days postpartum (D10), endogenous immune activity along the GI tract appears subdued, owing in part to low microbial presence, fewer mucosal immune cells, and indirect suppression by factors such as epidermal growth factor (EGF) in breast milk (22). However, GI changes begin to occur around D14, ultimately reaching their peak around D21 which coincides with the time of weaning. The microbial load in the colon increases exponentially during this time, particularly with respect to *Clostridia* and *Bacteroides* members (20). Hitherto, the ability of mucosal antigen presenting cells (APC) to sample and present luminal microbial antigen is inhibited due to the low permeability of the epithelial barrier and lack of trans-epithelial dendrites (22), but decreasing levels of murine breast milk EGF around D14 allows for the spontaneous formation of goblet cell associated antigen passages (GAPs). GAPs produce a transient permeability in the murine small intestine and colon, enabling mucosal APC to acquire and present microbial antigen which results in antigen-specific mucosal CD4^+^ T lymphocyte activation and proliferation. When these events occur from D14-D21 in mice, clonotypic expansion results in a population of CD4^+^ Foxp3^+^ regulatory T lymphocytes (Tregs) (20, 22), a subset of T lymphocytes that promotes antigen-specific tolerance. This tolerance appears to be maintained into adulthood. At the same time, the increase in microbial antigen exposure, concomitant with the reduction of inhibiting breast milk factors, induces significant changes in the transcriptional profile of the murine GI tract (20, 21). Upregulated expression of genes involved in barrier integrity, mucosal defense, chemotaxis, and inflammation are observed at D21, although they quickly revert to baseline levels by 4 weeks of age (20). Similarly, by 4 weeks of age colonic GAPs are largely gone and the impermeability of the epithelial barrier is restored (22).

Particularly enlightening is what ensues when the weaning reaction fails to occur, or occurs outside the D14-21 window of time. If microbial stimulation is unavailable prior to D21 (for instance, germ-free mice colonized post D21) or breast milk EGF remains elevated up until weaning, a phenomenon referred to as “pathological imprinting” occurs, such that mice become highly susceptible to inflammatory diseases in adulthood, showing dysregulation of a broad range of immune responses (20, 22). Pathological imprinting also occurs if mice are unable to respond to microbial stimulation due to genetic manipulation (22), are experimentally prevented from inducing CD4^+^Foxp3^+^ Tregs (20), or are weaned outside the D14-21 window (20). Each of these scenarios yields clonotypic expansion of CD4^+^ T lymphocytes that are pro-inflammatory in nature and tolerance is not established. Thus, a successful weaning reaction is reliant on a consortium of factors that include microbial presence prior to weaning, receding levels of breast milk EGF allowing for formation of colonic GAPs, the ability to induce CD4^+^Foxp3^+^ Tregs, and a significant dynamic induction of immune-associated gene expression around D21. Deviations from this protocol result in pathological imprinting that promotes dysregulated immune responses later in life (20, 22). Importantly, a suboptimal weaning reaction may not leave an obvious signature in adulthood; in the cited studies, no differences were observed in the microbiota or CD4^+^Foxp3^+^ Treg numbers of adult mice despite a suboptimal weaning reaction and subsequent immune dysregulation (20, 22).

The experimental and genetic manipulations utilized in animal studies are artificial and thus, their translational potential remains to be determined. However, these studies may help explain some of the observations in human disease. For example, the knowledge that the change in breast milk EGF concentration is responsible for the timing of GAP formation, and that manipulating the timing of GAP formation to occur earlier or later results in pathogenic imprinting might help explain why the exclusive use of formula or early use of cow’s milk are consistently associated with immunodysregulatory diseases in humans (23). The composition of formula and cow’s milk is static throughout infant development, abolishing the possibility for dynamic interaction with infant physiology in a time-dependent manner. Similarly, the observation that a strong weaning reaction and development of tolerance are dependent on early microbial stimulation may explain the negative correlation between inflammatory/autoimmune diseases and less-hygienic living conditions (non-industrialized countries, rural or farm living, pets, etc) (24), since early microbial exposure in these situations would likely be increased and more diverse.

Despite the phenomenal surge in endogenous immune growth during this time, infants remain highly susceptible to infection. Indeed, 3.1 million infants under 1 yr of age were admitted to hospital with an infectious disease in the United States between 2001 and 2014, with more than 6000 succumbing to infection (25). Thus, infant survival during this challenging time depends heavily on maternally provided immunity. While bolstering infant defense against these insults, these maternal provisions continue to act as an educator in the development and maturation of the infant’s immune system, leaving an indelible imprint in preparation for future immune insults.

## 3 Maternal effects on infant microbiota development

Environmental cues are sensed by microbial communities in the gut, a site that interfaces dietary nutrients, resident microbes and the host mucosal immune system (26). Intestinal homeostasis is maintained through cooperated actions of a mucus-lined physical barrier, and an immunological barrier consisting of epithelium- and immune cell- derived antimicrobial substances, proinflammatory cytokines and chemoattractants, and humoral effector molecules such as anti-commensal immunoglobulins (27). An imbalance in the gut microbiome, termed dysbiosis, causes altered generation of diet-dependent immunomodulatory metabolites (28) and can contribute to local and systemic inflammation and loss of immune tolerance (29, 30).

The microbiota and its role in health and disease has been an area of increasingly intense investigation over the last few decades. Both the findings of microbiome studies and their associations with T1D have been thoroughly reviewed by others (12–15), but the effects of dysbiosis during pregnancy and breastfeeding on the development of T1D in infants has received much less attention. In the following sections, we discuss maternal influences on the development of the neonatal microbiome and review the few studies that have addressed the effects of T1D associated maternal dysbiosis on infant disease development.

### 3.1 Origin and outcome of infant gut colonization

Although actively debated, it is generally accepted that babies *in utero* are largely sterile, resulting in a GI tract that is extremely vulnerable to microbial colonization at birth (31). Amidst the numerous factors that influence the development of the infant microbiota, mode of delivery and breastfeeding are amongst the most influential (32, 33). During a natural birth, microorganisms from the vaginal tract act as pioneer colonizers of this highly hospitable niche, resulting in an early infant microbiome that resembles the maternal vaginal microbiome. Because the vaginal microbiome is frequently dominated by *Lactobacillus* species (34), vaginally-delivered infants tend to show higher proportions of this genus (32, 35). For infants born *via* Caesarean section, the early microbiome resembles the maternal skin flora and as a group, show significant differences from vaginally delivered neonates (32, 35). However, by 6 months of age these differences have largely disappeared and infant diet (breastfed or formula-fed) emerges as the main stratifier of microbiome composition (36–38). Breast milk harbors its own unique microbiome which includes viable microorganisms from both *Lactobacillus* and *Bifidobacteria* genera in humans (39). Both vaginally-acquired and breast milk provided microorganisms aid in the early development of a healthy microbiome and diminish opportunity for pathogenic microbes to establish colonization (40–42). Human milk oligosaccharides aid in this endeavor by selectively promoting the growth of commensal bacteria, including *Lactobacillus* and *Bifidobacterium* species. *Bifidobacterium* accounts for a significant proportion of the microbiota in vaginally-delivered, breastfed infants after the neonatal period (43–45). The health-promoting effects of both *Lactobacillus* and *Bifidobacterium* have been well elucidated, with studies demonstrating their ability to inhibit enteric infections (46, 47), contribute to the colonization of other commensal species *via* cross-feeding (48, 49), and bolster production of the immunomodulatory short chain fatty acids (SCFA) (43, 44, 50). The production of SCFA, especially butyrate, is a key contributor to gut barrier function, as these metabolites are both an energy source for intestinal epithelial cells (IEC) and promoters of homeostatic, tolerogenic immune responses (43, 44). Moreover, reduced pathogen presence resulting from strong commensal growth serves to protect the infant from a variety of infections (33). Indeed, enough evidence has emerged with respect to the importance of *Bifidobacterium* during infancy to motivate a clinical trial in Europe exploring the protective effects of *B. longum* ssp. *infantis* administration to infants at risk of developing T1D (51).

Microbiome colonization plays a critical role in stimulating the neonatal immune system (40–42). Studies using germfree mice have provided a glimpse into the molecular and functional immune changes prompted by microbial colonization. Compared to pups born to germfree mice, those born to dams colonized with a complex microbiota showed upregulated ileal expression of lymphocyte activation genes (CD45, *Nfkb*) as early as postpartum day 1 (D1), followed by upregulated expression of genes involved in microbial sensing (*Tlr2, Tlr4*) and cytokine production (*Tslp, Tnf*) by D6 (52). By D23, the terminal ileum showed decreased expression of several chemokines (*Ccl2, Ccl3, Cxcl1, Cxcl2*), suggesting reduced trafficking of immune cells to the gut. At the same time point, genes involved in antigen presentation (CD80, *H2-Ea*) and lymphocyte activation (CD40) were downregulated in the mesenteric lymph nodes (MLN) (52). Using mice colonized postnatally, El Aidy et al. showed that introduction of a complex microbiota resulted in expression of innate immune associated genes (*Reg3g, Reg3b, Retnlb*) within 4 days of exposure, followed by increased expression of cytokines (IFNγ, TNFα, IL-10) and antigen presentation elements (*Tap1, Tap2, Psmb8, Psmb9*) by post-colonization D16 (21). Further evidence of gut-resident microbial stimulation of the immune system has been demonstrated by studies utilizing probiotics. Oral administration of various *Bifidobacterium* or *Lactobacillus* species has been shown to strengthen GI immune responses by promoting recruitment of innate immune cells, increasing their expression of microbial recognition receptors and enhancing their phagocytic activity, as well as stimulating intestinal IgA secretion (53). *Lactobacillus casei* strain DN-114001, when provided in a fermented milk product, has been shown to interact directly with both IEC and mucosal immune tissues in the small intestine, resulting in increased numbers of IgA^+^ B cells, CD4^+^ and CD8^+^ T lymphocytes, macrophages and goblet cells in adult BALB/c mice (54). Interestingly, when the same *L. casei* DN-114001-containing fermented milk product was administered to breastfeeding murine dams, their offspring exhibited an early transient reduction in small intestinal goblet cells, macrophages and dendritic cells, possibly due to the altered representation of microbial taxa observed during this period (55).

Microbial exposure has been shown to expand B and T lymphocyte repertoires, promoting lymphocyte proliferation and influencing their differentiation (20, 56–61). Trafficking of microbial antigen from the intestine to the thymus *via* dendritic cells results in the stimulation and proliferation of populations of microbe-specific T lymphocytes (57), including microbiota-specific Tregs that develop and differentiate in the thymus, subsequently migrating to the intestine (58). Additionally, upon early life exposure to the microbiota, peripheral T lymphocytes can be induced to a regulatory phenoytpe referred to as inducible Tregs (iTregs) which express the transcription factors Foxp3 and RORγt (62). Perinatal exposure to broad-spectrum antibiotics, which induces a state of dysbiosis, has been shown to significantly decrease the numbers of iTregs in the colon and contribute to dysregulated intestinal immune responses later in life (63), further demonstrating the ability of the microbiota to influence the adaptive immune response.

Overall, colonization stimulates both pro- and anti-inflammatory signals that together drive a balanced tolerogenic response in a time-specific manner, allowing for continued microbial presence and microbiome establishment while simultaneously promoting the maturation and expansion of the neonatal immune system. Perturbations of these microbially-influenced immune signals often result in irreparable impairment of the developing immune system.

### 3.2 Contributions of the early microbiome to health & disease

The importance of early establishment of a healthy, balanced microbiota has become increasingly evident. Correlative studies in humans consistently show increased prevalence of immunodysregulatory diseases amongst individuals exposed to dysbiosis-promoting events during infancy and early childhood (24). Similarly, studies in animals have demonstrated the deleterious effects of early dysbiosis on health status later in life and have provided substantial evidence for the health-promoting effects of SCFA-producing commensal microorganisms such as *Bifidobacterium* and *Lactobacillus* particularly during the gestational and neonatal periods (44). For example, SCFA administration to pregnant and breastfeeding dams has been shown to remodel the infant gut microbiome, alter lymphocyte populations in the gut, ameliorate inflammation in the pancreas, and reduce disease incidence in animal models of T1D (64, 65). Interestingly, this outcome cannot be achieved with SCFA supplementation after weaning alone (64), underscoring the importance of maternal provisions during this time period.

**Figure 1:**
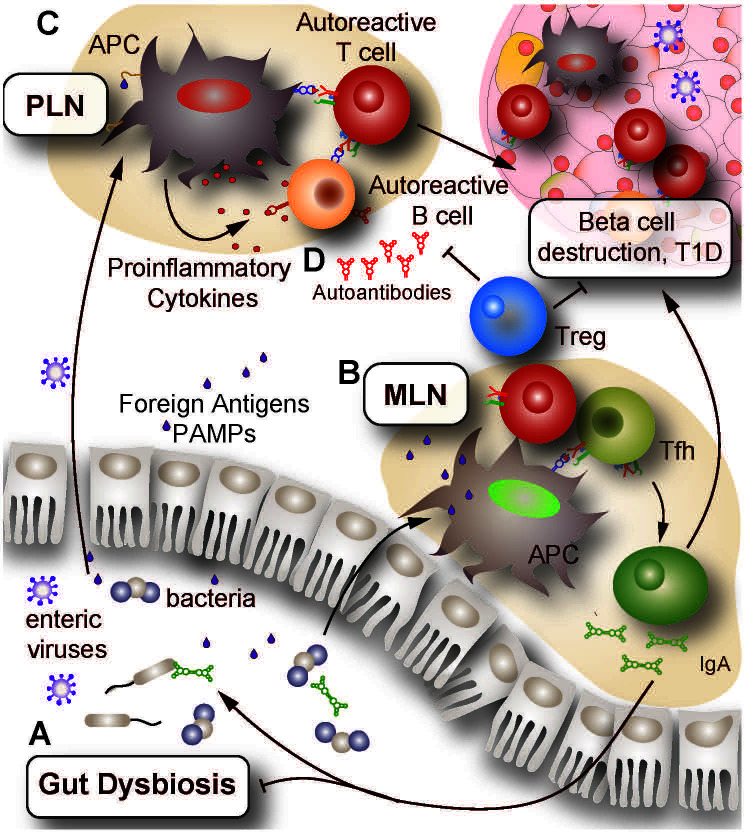
Proposed mechanisms of autoimmune activation leading to insulitis. **(A)** Environmental triggers such as infection or prolonged antibiotic use can induce changes in the GI tract leading to dysbiosis, increased GI inflammation and impaired barrier integrity. Together, these changes promote microbial translocation across the epithelial lining and increase PAMP presence in secondary lymphoid organs such as the MLN and PLN. **(B)** Presentation of PAMPs to T lymphocytes in the MLN and PLN results in T and B lymphocyte activation and promotion of a pro-inflammatory environment. Impaired central and peripheral tolerance in genetically at-risk individuals can allow for the erroneous activation of autoreactive lymphocytes by multiple mechanisms, including molecular mimicry and bystander effects. **(C)** Enteric viruses can gain access to pancreatic islets, resulting in infection and death of beta cells. As infected cells die and release autoantigens, APC internalize these autoantigens and present them to lymphocytes in the PLN. Owing to ineffective peripheral tolerance, islet-reactive lymphocytes become activated and infiltrate the islets, promoting further beta cell destruction. **(D)** Activated islet-specific B lymphocytes undergo differentiation to plasma cells which enter systemic circulation and secrete islet-specific antibodies into the bloodstream. Detection of these autoantibodies is a clinical indicator preceding diagnosis of T1D. GI, gastrointestinal; PAMP, pathogen associated molecular patterns; MLN, mesenteric lymph nodes; PLN, pancreatic lymph nodes; APC, antigen presenting cell; Tfh, follicular helper T lymphocytes; Treg, regulatory T lymphocyte.

In older individuals affected with T1D, an altered gut environment is frequently seen both prior to and following diagnosis. An aberrant microbiome has been observed to precede or coincide with disease onset (29, 45, 66–72) and is believed to be a contributor to disease development (**Figure 1**). Notably, a decreased abundance of SCFA-producing bacteria has been reported in children at various stages of T1D progression (29, 66, 69, 70, 73) and a decline in gut barrier function, as evidenced by increased permeability and microbial translocation, has been observed in patients (66, 74–77). Similar observations of dysbiosis and altered SCFA levels were made in animal models of T1D (78–80), and further supported by demonstration that the administration of health-promoting bacterial strains such as *Lactobacillus johnsonii* could delay or reduce T1D incidence in disease-prone rats (81). The disease-modifying role of the microbiome was further demonstrated *via* fecal microbiota transfer studies in mice (82, 83) and in a human clinical trial with new onset T1D individuals (84). Finally, dysbiosis-promoting enteroviral infections, particularly coxsackieviral infections, during childhood have been strongly correlated with T1D in various human studies (23, 85–91) (**Figure 1**). Taken together, these findings and observations strongly argue for the presence and importance of early maternal influences on intestinal homeostasis that serve to protect against disease in later years.

In order to appreciate the relevance of microbiome modifications observed in T1D, it is necessary to establish what a healthy, disease-resistant microbiota looks like, particularly during infancy. However, the factors contributing to microbiome development are diverse, dynamic, and likely still largely unknown. Microbiome composition is influenced by genetics, delivery mode, maternal seeding, diet, age, antibiotic use, infection history, and disease status (24). There are differences observed across geographical regions and with different living environments (73). A household with pets influences microbiome composition, as does the presence or absence of older siblings (24). Furthermore, microbial composition can look different between two individuals but be very similar with respect to functional composition. To further complicate the matter, analysis of early life microbiome composition is often limited by the length of the study follow up period; it is very difficult to know whether an infant can be categorized as a healthy control when his or her future disease status is unknown. Thus, it is not an easy feat to ascertain the composition of a “normal, healthy” infant microbiota. In light of this, perhaps the most suitable option is to compare the collective microbiota of populations with a comparatively low incidence of T1D to those harbored by individuals at risk of developing T1D. Employing this rationale, the DIABIMMUNE study (92) followed the microbiota development of ~1000 infants from Russia, Estonia and Finland from birth to 3 years of age. During the timeframe of the study, Finland exhibited a 6 fold higher incidence of T1D compared to Russia (93), allowing for a comparison of microbiota development in low-T1D-risk Russian infants versus elevated-T1D-risk Finnish infants. During the first year of life, low T1D risk (aka “a healthy microbiota”) was associated with increased representation of the phylum Actinobacteria, specifically members of *Bifidobacterium* genus. Because of this, Russian infant microbiomes exhibited lower compositional and functional diversity up to 12 months of age, but by 3 years of age, had surpassed the diversity of Finnish infant microbiomes. With the exception of *Bifidobacterium* members, Russian infants showed a more plastic microbiome characterized by less strain stability during the first year of life. In contrast, Finnish infants had elevated levels of *Bacteroides* species, most notably *Bacteroides dorei*, with relatively stable strain persistence. The differences observed between infants from the two regions could not be accounted for by breastfeeding prevalence, and were instead attributed to disparities in environmental and living conditions between the two countries.

Some studies have attempted to elucidate an early life T1D-associated microbiota by analyzing infant microbiota samples and retrospectively categorizing them as T1D or healthy controls after following each participant for a given length of time. The length of the follow up period can be a significant limiting factor, however, and often accounts for discrepancies observed between studies. In order to minimize this limitation, the presence of T1D associated AA is often used as a surrogate for T1D incidence, as AA are often present during the preclinical disease period. Applying this methodology, The Environmental Determinants for Diabetes in the Young (TEDDY) Study (45) found that infants from 3 - 14 months of age had a microbiota dominated by one of three *Bifidobacterium* species and thus showed low diversity, regardless of AA presence or subsequent T1D status. Geographical region and provision of breast milk were the two major factors accounting for differences between the infants, and cessation of breastfeeding was associated with an increase in the phylum Firmicutes in all infants. Interestingly, maternal T1D status played almost no part in microbiota stratification at any age. Differences observed between the T1D-affected individuals and those identified as healthy controls were subtle and included elevated *Parabacteroides* and Prevotellaceae representation, along with decreased presence of *Dialister*, *Akkermansia* and Ruminococcaceae members. In accordance with this, the DIABIMMUNE study (92) noted rising *Dialister invisus* levels by 24 months of age in the Russian cohort compared to the Finnish infants. In contrast, *Dialister invisus* was found to be a T1D-associated species in studies analyzing microbiomes of older participants (12, 68), underscoring the importance of distinguishing age-associated from disease-associated microbiome differences. The role of *Akkermansia municiphila* in regulating disease has been demonstrated in animal studies, where it has been found to lower T1D incidence in NOD mice (82). *Prevotella* is a genus that often emerges as differentially represented in T1D, although studies are in disagreement over the direction of change (12). This is possibly due to differences in age or geographical location of the participants, or are reflective of changes at different stages of T1D development. Thus, these discrepancies may distinguish microbial modifications that drive autoimmunity from those that are a consequence of disease.

### 3.3 Contribution of maternal dysbiosis to T1D

There is evidence that maternal dysbiosis may facilitate early infant dysbiosis, beginning even prior to birth. Studies analyzing the microorganisms in meconium of neonates have found that maternal health status affects the early gut microbiome of infants. Compared to those from healthy mothers, infants born to hyperglycemic mothers harbor a microbiome exhibiting reduced metabolic potential, increased bacterial diversity, altered representation of bacterial phyla with reduced *Prevotella* and *Lactobacillus* members, and increased presence of eukaryotic viruses (94–96). Furthermore, the altered gut metabolome observed in hyperglycemic mothers is reflected in the blood and fecal metabolome of their infants at birth, particularly with respect to metabolites involved in biotin metabolism (97). This is unlikely to be related to vaginal microbial transfer or breastfeeding, as meconium is passed within the first few hours to days after birth, and instead may reflect events occurring *in utero*, lending credence to the *in utero* colonization theory (31). It is worth noting that these studies focused on hyperglycemic mothers presenting with either type 2 or gestational diabetes, so whether these findings are relevant in T1D is as yet unknown.

Since the vaginal microbiota is a significant source of microorganisms for establishment of the early microbiome in vaginally-delivered infants, it is noteworthy that maternal vaginal dysbiosis is associated with both maternal hyperglycemia (95) and T1D incidence in offspring (34, 98). In a study of pregnant women with gestational diabetes, increased blood glucose levels corresponded with high *Prevotella*/*Aerococcus* and low *Faecalibacterium*/*Fusobacterium* ratios in both the vaginal and intestinal tracts. Furthermore, these same trends were observed in the meconium of their infants (95). In studies aimed at elucidating the association between child T1D and maternal vaginal dysbiosis, both Tejesvi et al. (34) and Ruotsalainen et al. (98) reported maternal vaginal microbiomes dominated largely by the genus *Lactobacillus*, regardless of offspring health status. However, in women that had previously given birth to a child diagnosed with T1D, the proportion of *Lactobacillus* was reduced slightly but significantly, resulting in greater microbial diversity overall (34, 98). Specifically, they found increased prevalence of the bacterial genera *Aerococcus* and *Prevotella*. Furthermore, these women showed alterations in vaginal fungal communities, exhibiting an increase in both incidence and abundance of the fungal genus *Tylospora* (98). A T1D associated altered vaginal mycobiome is of interest, since children with T1D associated AA have been shown to exhibit increased presence of *Saccharomyces* and *Candida* along with a reduction in *Verticillium* genus members (99). Although very enlightening, the studies by Ruotsalainen et al. (98) and Tejesvi et al. (34) were conducted years after childbirth, so the relevance of vaginal dysbiosis to disease and its effect on infant microbiome development is unknown. Thus, studies exploring maternal vaginal dysbiosis prior to or following birth, along with exploration of microbiome development and later disease incidence in their infants, would enhance our understanding of the relationship between the two.

### 3.4 Effects of maternal dysbiosis during breastfeeding

An altered intestinal microbiome in mothers with T1D has further implications for breastfeeding. Human milk is populated, at least in part, with microorganisms and metabolites from the gut microbiome (100), raising the possibility that breastfeeding by affected mothers could promote the development of an unbalanced infant microbiota. Although we were unable to find studies addressing this question in T1D, there is evidence that maternal health status can affect breast milk composition. One study observed altered human colostrum microbiome composition in breastfeeding mothers with gestational diabetes, noting increased diversity and an over-representation of several genera, including *Prevotella* (101). In an animal study, vancomycin-induced dysbiosis in pregnant and breastfeeding mice resulted in increased immunoglobulin in breast milk and contributed to an increase in splenic lymphocytes in the pups, particularly with respect to follicular and marginal zone B cells. These changes were attributed to alterations in maternal gut microbiome activities, as vancomycin is not absorbed across the intestinal wall and so can only act locally (102). The long term consequence of the altered splenic lymphocyte populations was not explored, so whether vancomycin-induced maternal dysbiosis and the resulting increase in breast milk immunoglobulin proved protective or pathogenic in nature is unclear. Indeed, the possibility that a dysbiotic maternal microbiota, along with the ensuing alteration of the infant microbiota and heightened GI immune response, may have beneficial consequences to infant health is a novel idea, but one that is gaining merit and thus compels consideration. Usami et al. demonstrated the ability of *Bacteroides acidifaciens* and *Prevotella buccalisto* to elicit strong humoral responses in the Peyer’s Patches (PP) of lactating mice (103). The resulting IgA-secreting plasma cells migrated to the mammary glands, where their IgA production contributed significantly to the milk IgA concentration. In comparison to milk IgA produced in the absence of intestinal *P. buccalisto* and *B. acidifaciens*, the increased milk IgA was found to more efficiently coat fecal microorganisms, providing a potential mechanism for maternal protection against *P. buccalisto* and *B. acidifaciens* overgrowth in offspring. Furthermore, given that T1D dysbiosis is frequently associated with increased *Prevotella* and *Bacteroides* species (12), this is evidence that a dysbiotic maternal microbiome featuring elevated numbers of immunogenic commensals may contribute to infant protection *via* the induction of microbe-specific IgA production secreted in milk. Another example of the potential health benefit of neonatal intestinal inflammation can be found in a study by Valladares et al. (81). The authors demonstrated that the daily administration of *Lactobacillus johnsonii* to T1D-prone rats during adult life increased expression of the tight junction protein claudin 1 and decreased interferon (IFN)-γ levels in the ileum. The resulting health benefit was reduced disease incidence. Surprisingly, administration of the same *L. johnsonii* strain to rats pre-weaning did not result in disease amelioration, but instead marginally increased the disease incidence. The authors proposed that this unexpected result was a product of heightened neonatal stress from the administration procedure (daily oral gavage), suggesting that the stress outweighed the later health benefits of the bacterium (81). However, in considering the importance of the heightened immune activity that constitutes the weaning reaction, an alternative explanation is that reducing or inhibiting pro-inflammatory responses by administration of *L. johnsonii* during the pre-weaning period yielded an overall health deficit in the form of immune dysregulation, which promoted T1D development within the disease-prone animals.

In humans, breastfeeding has been studied extensively for its role in conferring protection from T1D. Although the strength of the correlation differs between studies, the consensus is that infants breastfed for 3 or more months show a reduced incidence of T1D later in life (104, 105). The reasons for this, and the mechanisms behind it, are likely multi-faceted and undoubtedly include microbiome modification. In both human studies and animal models of T1D, investigations into GI alterations have yielded insights into possible mechanisms that link breastfeeding and the neonatal microbiome with disease. In humans, exclusive breastfeeding was shown to correlate with increased populations of Tregs in GI tracts of 3 week old infants and was associated with increased presence of *Veillonella* and *Gemella*, known for their SCFA production, in stool samples (59). In mice, compared to diabetes-resistant strains, weaned non-obese diabetic (NOD) mice display alterations in microbiome composition accompanied by reduced small intestinal goblet cell numbers and diminished mucus secretion along the GI tract (106). The presence of a thick mucus layer along the GI tract is an essential part of the physical barrier maintaining separation of the microbiota from the gut epithelium, and deficits in this mucosal layer have been associated with inflammatory bowel diseases and gut dysbiosis (27, 107). Interestingly, when NOD pups were fostered to a diabetes-resistant dam immediately after birth, these impairments were not observed (106). Given these findings, it is possible that breast milk from a disease-resistant dam, which presumably differs in composition from NOD breast milk, is able to correct inherent GI deficits in disease-susceptible infants. Alternatively, GI alterations may develop in the pups as a result of breast milk from the NOD dam and thus are avoided completely by nursing from the foster dam. Whether it is the breast milk microbiome of the two differing dams giving rise to different microbiome development in the pups and subsequently affecting barrier function, or another aspect of the breast milk altogether, is an important question that wasn’t explored in this particular study, nor was the effect of fostering on T1D prevalence in the pups. Regardless, this study highlights the ability of breast milk to affect development of the neonatal GI tract, providing some mechanistic possibilities behind the negative correlation that exists between breastfeeding and T1D incidence in humans. Moving forward, the diabetogenic versus protective potential of breast milk from affected mothers is an important issue to address, as the increasingly popular establishment of human milk banks makes it possible to provide alternate sources of breast milk for infants at risk.

## 4 Maternal antibodies acting in the intestinal tract

For the first month of life, maternally derived antibodies in breast milk provide the bulk of gut immunoglobulin as the infant has yet to produce sufficient amounts of his or her own (108, 109). Even beyond this period, breast milk antibodies supplement gut immunoglobulin to a substantial degree (109). Breast milk contains antibodies of all isotypes but is particularly high in IgA, IgM and IgG (41). Pathogen-reactive IgG, produced either in response to maternal infection or arising as natural antibodies from exposure to maternal gut commensal species, can act in the gut lumen to coat enteric pathogens such as *Citrobacter rodentium* and enterotoxigenic *E. coli*, protecting the infant from infection (110, 111). Similarly, IgA and IgM accomplish their function in the gut lumen but tend to show less specificity for their targets, instead binding to microbial cell surface structures that are common between a number of microorganisms (56). Together, luminal antibodies protect against pathogen overgrowth and coat toxins and commensal microbes, keeping them away from the intestinal lining and establishing the spatial gradient of microbial density indicative of a healthy gut environment (112). Breast milk antibodies have been implicated in the maintenance of a tolerogenic environment unique to the neonatal intestine, helping to suppress CD4^+^ T lymphocyte maturation (113). Moreover, maternal IgA acts as an opsonin, promoting the phagocytosis of IgA-bound microorganisms by colostrum/breast milk phagocytes and activating their various immunological functions (114). This particular role for IgA may be species-dependent, as murine phagocytes do not express an Fc receptor for IgA (115).

T1D has been shown to complicate the delivery of breast milk antibodies to babies. In general, mothers with T1D have later onset of lactation and lower milk supply throughout the breastfeeding period, difficulties that are further exacerbated with poor blood glucose control (116). Additionally, hyperglycemia may lower colostrum IgA and IgG levels (117). Colostral phagocytes show significantly reduced activity in the presence of microbes lacking IgA opsonization (114), raising the possibility that decreased IgA levels in colostrum could impede gut immunity during the first weeks of neonatal life. Whether this is actually the case remains to be investigated. One study reported that IgA levels in T1D colostrum, though decreased, remained high enough to maintain substantial phagocytic activity in breast milk-resident phagocytes (114). Because very few human studies have looked at immunoglobulin content in breast milk from women with T1D (118), it is currently unclear whether antibody levels in breast milk are affected beyond the colostrum stage, although there is some support for decreased IgA in breast milk of hyperglycemic mothers (119). Furthermore, reduced IgA coating of fecal material is frequently reported in T1D, including studies using newly-weaned NOD mice (106), although it is unclear whether this is due to a decrease in endogenous IgA production or a deficit in breast milk immunoglobulin. The potential cause-and-effect relationships of intestinal dysbiosis and IgA responses in the setting of T1D is an area under active investigation in our laboratory. We hope this will shed light on a topic that has important implications in maternally-transmitted, non-genetically encoded mechanisms of disease susceptibility and resistance.

## 5 Active transport of maternal IgG – the FcRn

**Figure 2:**
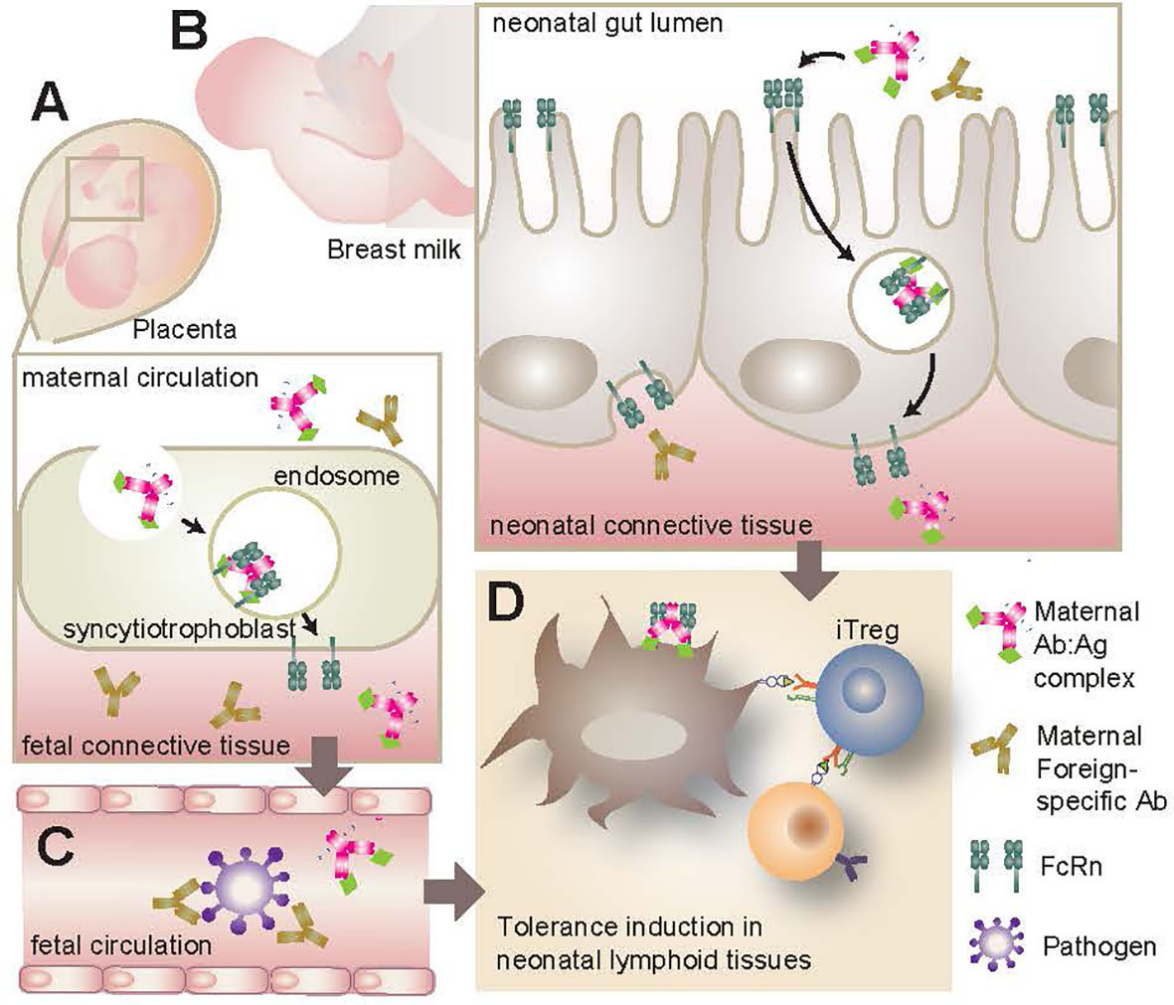
FcRn transport of maternal IgG. **(A)** FcRn expression in the placenta allows for the active transport of IgG from the mother’s bloodstream to the infant during gestation. If maternal IgG has bound antigen (Ab-Ag complex) this antigen will be transported to the infant as well, resulting in early antigenic exposure. **(B)** FcRn expression along the GI tract of the neonate can bind and transport maternal IgG and Ab-Ag complexes from breast milk across the intestinal barrier. These maternal antibodies can infiltrate local lymphoid tissues such as the MLN and PP. **(C)** Whether originating from maternal bloodstream or breast milk, IgG and Ab-Ag complexes can enter the infant’s circulation. Maternal IgG provides a source of passive immunity for the infant, neutralizing pathogens that would otherwise present a serious challenge for the underdeveloped neonatal immune system. **(D)** Maternal IgG and Ab-Ag complexes in infant circulation can gain access to various lymphoid organs where they can be taken up by APC *via* cell surface FcRn. Once endocytosed, the IgG can be recycled and the antigenic peptide presented to naive T lymphocytes. This early antigenic exposure generally results in a tolerogenic response, including the polarization and proliferation of CD4^+^ T lymphocytes to Treg. FcRn, neonatal Fc receptor; GI, gastrointestinal; MLN, mesenteric lymph nodes; PP, Peyer’s Patches; APC, antigen presenting cells; Treg, regulatory T lymphocytes.

The presence of a placental immunoglobulin transporter was first confirmed in humans in 1990 (120, 121) and was termed the neonatal Fc receptor (FcRn). Since then, its various roles in antibody transport, and more generally in fetal and neonatal immunity, have been investigated (**Figure 2**). Expression patterns of the FcRn are regulated both temporally and spatially. Age and tissue specific expression of FcRn allows for maternal antibody transfer both during fetal development across the placenta, termed placental transcytosis, and during early life through the uptake of antibodies in breast milk. Expression patterns of the FcRn differ between mice and humans due in part to being regulated by different promotors (122–124). In mice, FcRn expression is found in the epithelial cells of a number of tissues, including those of the yolk sac during gestation (125, 126). Additionally, IEC show FcRn expression up until weaning, after which expression on the apical surface is largely lost (110, 127) although there is evidence of retained expression on the basolateral surface (110). The majority of murine maternal immunoglobulin is transferred *via* breast milk ingestion during the first 21 days of life (110, 122). This transfer of breast milk immunoglobulin is largely restricted to IgG, since FcRn shows strong specificity for binding and transport of IgG with little to no affinity for other immunoglobulin isotypes (124, 128–130). In contrast, human babies acquire their bulk of maternal IgG *via* placental transcytosis (122). Human placental endothelial cells and syncytiotrophoblasts express FcRn (126, 131–135) allowing for significant transfer of maternal antibodies particularly during the third trimester (136–138). Similar to mice, human FcRn is also expressed by IEC to allow for breast milk IgG uptake; this expression remains well into adulthood. Furthermore, the presence of FcRn on various murine and human hematopoietic cells, particularly APC, provides a mechanistic role for maternal IgG in fetal and neonatal immune development (126, 128, 139, 140).

The expression of FcRn by these various tissue types fulfills a number of roles with respect to humoral immunity and immune development during infancy. The active transport of maternal IgG from mom to infant provides a source of systemic passive immunity against pathogens and is highly effective in protecting the neonate against infection and disease (141, 142) so much so that it has become common practice to vaccinate pregnant women against infections such as influenza, measles, mumps and rubella with the goal of reducing infection in their babies (143, 144). Furthermore, the binding and transport of immunoglobulin by FcRn results in recycling of the antibody and extension of its half life (145), extending its immune potential in the infant. Since viral infections *in utero* and during the neonatal period have been associated with increased risk of developing T1D in childhood (85, 87–90), the antiviral protection afforded to offspring *via* FcRn delivery of maternal antibodies suggests a possible link between the function of FcRn and T1D risk in genetically susceptible individuals. Moreover, the presence of FcRn on the surface of phagocytic cells and APC facilitates the capture of maternal IgG-antigen (Ab-Ag) complexes and subsequent presentation of maternally-derived antigen to naive T lymphocytes (122, 146). This has numerous implications for fetal immune development and its role in disease will be discussed at length in the following sections.

Chronic hyperglycemia has been shown to have adverse effects in pregnancy, including a decrease in maternal serum antibody levels despite a sufficient population of B lymphocytes (117, 147). Furthermore, there is evidence that FcRn expression by placental and cord blood cells is reduced under hyperglycemic conditions (147), although the effect that this has on transplacental IgG transfer is unclear: the rate of transfer may be decreased but overall cord blood IgG levels are higher than those seen under normal glycemic conditions, suggesting increased transplacental IgG transfer (117, 147). Although further clarification is needed, it remains that hyperglycemia alters several factors in the process of maternal antibody transfer and therefore may contribute to altered maternal antibody acquisition for the infant.

## 6 Maternal antibodies in immune development

The pre- and postnatal transfer of IgG antibodies from mom to infant is a well documented phenomenon that occurs in every immunocompetent murine and human pregnancy. It is less clear, however, what role these antibodies play and what effect they have on the developing neonate, particularly within the context of autoimmune diseases. Exposure to antigen is required in order to educate and expand immature leukocyte populations and diversify lymphocyte repertoires in the neonate (56, 59, 60). Maternally acquired IgG plays an active role in the transport of antigen to the neonate, delivering maternal Ab-Ag complexes *via* the FcRn across the placenta. After birth, FcRn expression on infant IEC allows uptake of maternal IgG from breast milk, which can then have systemic effects. Maternal IgG can be detected in the MLN and PP of murine offspring by two weeks of age and may allow for the uptake of commensal antigen by way of Ab-Ag complexes binding to the FcRn on immune cells, thereby increasing microbial antigen sampling and presentation (148). By and large, this mode of antigen transfer induces a tolerogenic response in the neonate by promoting the proliferation of CD4^+^ Treg populations. This has been demonstrated in several mouse studies using ovalbumin (OVA) challenge, showing that postnatal transfer of OVA-IgG complexes in breast milk of OVA sensitized dams resulted in the proliferation of OVA-specific Treg populations and tolerance to subsequent OVA challenge in the pups (149–151). The mechanism involves the capture of Ab-Ag complexes by CD11c^+^ dendritic cells *via* cell surface FcRn and subsequent transport to the thymus for presentation to naive T lymphocytes (151). This phenomenon has been exploited as a potential therapeutic treatment for islet autoimmunity in high risk infants. Culina et al. (152) proposed that fetal introduction of preproinsulin (PPI), a common autoantigen in T1D, could result in the induction of tolerance to PPI and thereby reduce disease incidence in high risk neonates. They created a PPI peptide fragment fused to the Fc portion of IgG molecule (PPI-Fc) and administered it to pregnant NOD dams as well as pregnant transgenic dams that harbor PPI-specific CD8^+^ T lymphocytes. This fusion protein was transplacentally delivered to the embryos *via* placental FcRn and resulted in an increased number of thymic-derived FoxP3^+^ Tregs, impaired PPI-specific CD8^+^ T cell-mediated cytotoxicity in the transgenic offspring and reduced T1D incidence in the NOD offspring (152). Intriguingly, when a follow-up experiment was conducted using oral administration of the same PPI-Fc molecule to pups one day postpartum, almost none of these tolerogenic effects ensued despite confirmed FcRn-dependent PPI-Fc uptake in the intestine and APC-mediated delivery to the thymus (153). These data strongly suggest that transplacental delivery of maternal IgG induces physiological events that differ from those that occur when the same antibodies are acquired through breast milk. Whether this is due to route of administration, age at acquisition, or alternative unexplored factors is currently unknown. Regardless, this is evidence of numerous factors at work during this short but crucial period of time and further exploration of these events will enable us to gain a better understanding of the role of maternal antibodies in neonatal immune development.

### 6.1 Foreign antigen-specific maternal antibodies in T1D

Given the extensive evidence linking enterovirus infections to diabetogenic events in both humans and animal models of T1D (87, 91), including maternal enteroviral infections during pregnancy (60) and a positive correlation between circulating maternal enteroviral antibodies and islet AA (154), it is counterintuitive to learn that there is a negative correlation between regional frequencies of enteroviral infections and incidence of T1D throughout the world (155). Furthermore, this same inverse relationship exists between maternal serum enteroviral antibodies during pregnancy and regional T1D incidence in the population (156); T1D prevalence is higher in populations where women of childbearing age harbor low serum levels of anti-enteroviral antibodies. A hypothesis for this apparent paradox was put forward by Viskari et al. (156) who postulated that enterovirus associated beta cell autoimmunity could be considered a complication of enteroviral infection that arises due to increased viral susceptibility in an individual. This theory is supported by the observation that some enteroviruses, such as Coxsackie B4 virus, show a tropism for pancreatic beta cells and can promote islet-specific autoimmunity *via* the release of previously sequestered antigen following beta cell damage (157) (**Figure 1**). One plausible explanation for increased viral susceptibility is a lack of maternally derived and transferred enteroviral neutralizing antibodies during gestation in regions of low enterovirus epidemiology, resulting in a lack of passive protection for the neonate (156). This hypothesis was experimentally investigated using Coxsackie B virus infection in animal models of T1D (158). The authors of the study demonstrated that infection of female mice generated serum neutralizing antibodies that, upon subsequent breeding, were transferred to the offspring and could be detected in serum at weaning. Furthermore, these maternally derived antibodies conferred protection against subsequent Coxsackie B virus infection in the offspring, and resulted in reduced beta cell autoimmunity and T1D development. These experimental results are supported by the observation that although Coxsackie virus B1 (CVB1) exposure elevates risk of developing islet autoimmunity in genetically at risk children, those harboring maternal neutralizing antibodies against CVB1 at birth have some attenuation of that risk (159).

### 6.2 Collaboration between the maternal microbiome and maternally-derived immunoglobulin

As discussed by Larsson et al. (158), the Coxsackie B studies mentioned above lend support to the ‘hygiene hypothesis’, a broader school of thought that postulates the amplified incidence of autoimmune diseases experienced in industrialized regions of the world is attributable to the decreased microbial exposure and challenge inherent in ‘cleaner’ societies. The relevance of this theory to the current review becomes apparent when one considers the tremendous amount of research showing the importance of maternally provided passive immunity; since mom cannot pass on to her babe that which she doesn’t have, reduced maternal exposure to microbes directly affects the immune health, development and function of her child in a lifelong fashion. Furthermore, the implications of the hygiene hypothesis become evident when one considers the role of natural maternal antibodies; that is, antibodies present in the mother that are generated without known exposure to a pathogen or vaccination. Recent studies have established that microbiota-reactive IgG antibodies (AKA natural antibodies) are present in the serum of murine dams at steady state (110, 148). These anti-commensal IgG are conferred to the offspring primarily *via* the breast milk and can be detected in the gut lumen and serum (110, 148) as well as the MLN and PP (148) at 1-2 weeks of age. Koch et al. (148) showed that their absence, in conjunction with a deficiency of maternally acquired IgA, resulted in a compensatory reaction in the offspring characterized by an increase in both numbers and activation state of mucosal T follicular helper (Tfh) cells, germinal center B lymphocytes and CD4^+^ T effector cells at 25 days postpartum. Although this phenotype was transient, it is evidence of altered mucosal immune responses during an important stage of development and bears implications for future antigenic challenge and possible aberrant immune responses in the gut. Additionally, the authors found that lack of maternal IgG led to increased microbial translocation and presence in the MLN of offspring, a finding that holds relevance for T1D pathogenesis; increased microbial translocation across the epithelial barrier and movement to the MLN and pancreatic lymph nodes (PLN) is hypothesized to contribute to early events leading to the erroneous activation of autoreactive T lymphocytes (74, 75) (**Figure 1**).

Collaboration between the maternal microbiota and IgG transfer to the neonate can bolster offspring gut integrity. Using mouse models, Gomez de Aguero et al. (160) discovered that transient colonization of germfree dams during pregnancy resulted in changes in the small intestinal environment of the neonate after birth when compared to offspring from dams maintained under germfree conditions. Pups from transiently-colonized dams showed an increase in type 3 innate lymphoid cell (ILC3) and F4/80^+^CDllc^+^ cell numbers, along with an altered ileal transcriptome indicative of amplified cell proliferation, bile acid metabolism, immune function and epithelial barrier integrity. In line with this, they found improved gut barrier function in two week old offspring. The mechanism behind this phenotype was found to be the result of microbial metabolite delivery from dam to pup. Specifically, microbially-derived AhR ligands from the dam were being provided to the pup in part *via* maternal Ab-Ag complexes. Interestingly, this phenomenon was initiated *in utero* and continued during the postnatal period possibly *via* Ag-Ab complexes in the breast milk despite the fact that the dams had returned to a germfree status before giving birth. This study highlights the importance of the maternal microbiota to offspring health and development during the prenatal period.

### 6.3 Maternal autoantibodies in T1D

The acquisition of maternal antibodies has additional implications in the context of autoimmunity and the transfer of AA. In a number of diseases, the transplacental delivery of maternal AA has deleterious effects on the health of the baby. For instance, AA raised against ribonucleoprotein particles SS-A/Ro and SS-B/La in a mother with Sjogren’s syndrome can have devastating effects when transmitted to an infant during pregnancy, leading to outcomes of neuropsychiatric impairment, liver damage and congenital heart block (122, 161). Similarly, mothers suffering from myasthenia gravis or thrombocytopenia purpura frequently transfer AA during pregnancy, resulting in increased incidence of disease in their babies (146). Arguments for a pathogenic role of maternal AA in T1D have been put forth in a study using B cell deficient NOD mice. This study showed that maternally derived IgG persisted in serum past 25 weeks of age and insulin AA were detectable until 5 weeks of age. The complete elimination of B cells, and thus both pre- and postnatally acquired maternal AA, as well as the selective elimination of AA through early embryo transplant to a non-autoimmune strain of mouse, significantly reduced disease incidence in NOD offspring (162). However, a second study in NOD mice reported contradicting findings in pregnant dams that had been immunized with insulin (IAA^+^) or vehicle control (IAA^-^). The authors found that the presence or absence of maternal insulin AA had no bearing on subsequent islet autoimmunity and T1D development in their progeny despite confirmed transfer of insulin AA from IAA^+^ dams to the offspring (163), suggesting that high maternal insulin AA levels do not contribute to the development of T1D in offspring. This conclusion is corroborated in studies in humans where evidence supporting a pathogenic role for maternal T1D associated AA is lacking (1–11).

Studies on familial T1D have found that among children with a parent with T1D, those with an affected mother show up to 4 fold lower incidence of disease than those with an affected father (1–11) while only few reports support the opposite (164). This discrepancy may be due in part to the length of the study follow up period. The German BABY-DIAB study (165) analyzed blood samples from more than 1000 children born to a parent with T1D (67% mother, 33% father). Of the children with affected fathers, all were negative for AA at birth. In contrast, >75% of the neonates born to affected mothers harbored circulating AA. The AA were deemed to be of maternal origin since they were of the same specificity and isotype as those detected in the mothers at delivery. Indeed, a consistent finding in many human studies is a positive correlation between maternal AA levels and those measured in cord blood or neonatal circulation at birth, indicating significant transplacental transfer of T1D associated AA. Furthermore, most studies report the elimination of maternal AA by 9 months of age (164–167). In the German BABY-DIAB study (165), only 12 children remained positive for T1D associated AA by 2 years of age. Interestingly, these same 12 children had exhibited amongst the lowest cord blood AA levels at birth. The ‘Trial to Reduce IDDM in the Genetically At Risk’ (TRIGR) study in Finland also noted a positive correlation between maternal and neonatal AA levels in their study of 74 T1D mothers and their newborns, although they found no evidence of induction of beta cell autoimmunity during fetal development (167). Thus, in spite of significant AA transfer, a causal link between maternal AA and infant T1D development has not been established.

As Giacoia (146) outlines in his review of AA in pregnancy, there are several factors that may influence the pathogenicity (or lack thereof) of maternally derived AA in the developing offspring. If the organ, cell or autoantigen in question is not yet well developed at the time of transfer, maternal antibodies may lack a target, rendering them fairly harmless. Similarly, if the autoantigen is inaccessible due to location within the organ or cell, pathogenicity may be reduced. Another factor is the status of immune development within the baby. Ab-Ag complexes often work by initiating complement activation, antibody dependent cellular cytotoxicity (ADCC) and antibody dependent cellular phagocytosis (ADCP). Successful complement activation, for example, requires all components of the complement cascade to be functional and present at high enough concentrations to be effective. Although some components are synthesized as early as 18-20 weeks gestation, many remain below 50% of adult levels even at full term (168, 169). Similarly, ADCC and ADCP rely on various lymphoid and myeloid cells to be functional and activated. Given the immature state of the fetal immune system, coupled with the aforementioned tolerogenic environment and potential lack of available antigen, it is possible that these antibody-mediated actions are largely absent when maternal AA are delivered transplacentally.

The transfer of maternal AA is not limited to pregnancy, raising questions about the effects of breastfeeding a newborn when the mother harbors T1D associated AA. Given that most T1D associated AA are of the IgG isotype, FcRn expression in the neonatal small intestine allows for potentially significant uptake of these maternal AA by the nursing baby. Interestingly, the majority of studies looking at the effects of breastfeeding on T1D development support a protective role for long-term breastfeeding (104, 105), although very few take into account the T1D status of the mother. Of those looking specifically at breastfeeding by affected mothers, we were unable to find any that supported a positive correlation between breastfeeding and disease development in offspring, and some evidence that supports an inverse relationship. The German BABY-DIAB study found no correlation between breastfeeding duration by affected mothers and AA levels in their infants (170), and reported a protective effect of breastfeeding for >3 months (1). Because of the lack of evidence linking breastfeeding by affected mothers to any deleterious effects in their infant, exclusive breastfeeding remains the recommendation of most health authorities (171–173).

### 6.4 Idiotypic/Anti-idiotypic network

**Figure 3:**
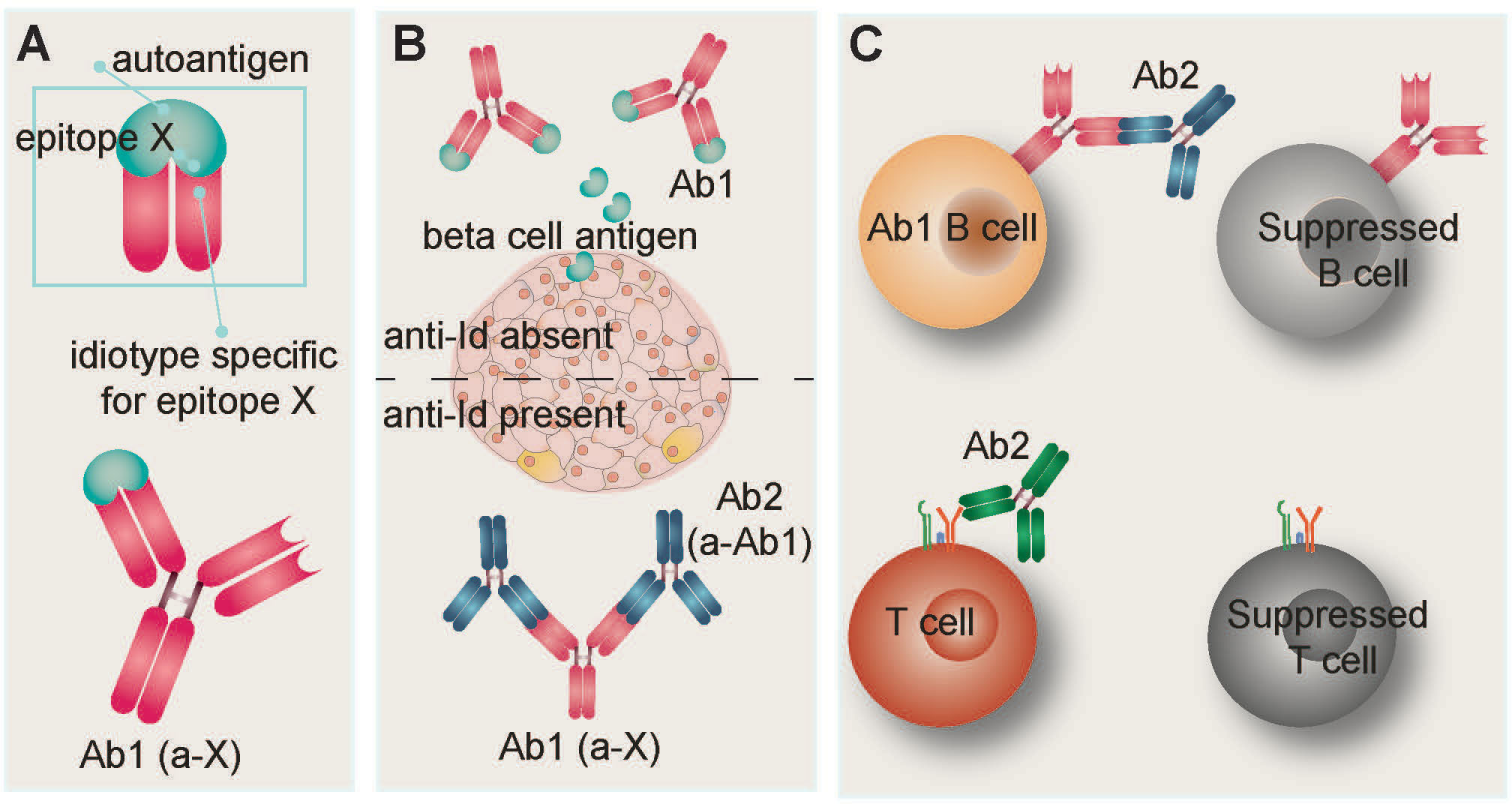
Idiotypic/anti-idiotypic network hypothesis. **(A)** The antigen binding site of an antibody is referred to as the idiotope. Idiotypic antibody Ab1 recognizes and binds epitope X on autoantigen X. In T1D, autoantigen X is frequently a beta cell protein such as insulin, glutamic acid decarboxylase (GAD), or tyrosine phosphatase-related insulinoma-associated 2 molecule (IA-2). Detection of T1D-associated autoantibodies is routinely used as a diagnostic indicator of disease. **(B)** The idiotypic network postulates that antibodies can be raised against the idiotope of Ab1. In the context of autoimmunity, these anti-idiotypic (anti-Id) antibodies (Ab2) compete with autoantigen X for binding sites on Ab1, resulting in Ab1 neutralization and limiting antibody-mediated damage to target cells. Anti-Id antibodies have been detected in healthy individuals and T1D patients, but are not routinely measured. **(C)** During the neonatal period, binding of anti-Id Ab2 to the B cell receptor of Ab1-bearing B lymphocytes leads to their suppression. Similarly, anti-Id antibodies can suppress T lymphocytes *via* binding to their T cell receptors.

An intriguing aspect of peripheral tolerance and immune regulation specifically pertaining to the humoral response is the proposed existence of an ‘idiotypic network’, a hypothesis that presents the immune system as a network of interconnected lymphocytes and antibodies that act to regulate the humoral arm of the adaptive system (174). The antigen recognition site of an antibody - the idiotope - fulfills its primary role in recognizing and binding to antigen, but can also function as an epitope for other antibodies. These idiotope-reactive antibodies - that is, antibodies that target and bind the idiotope of the original antibody - are termed anti-idiotypic (anti-Id) antibodies and according to the idiotypic network theory, act as a mechanism of peripheral tolerance to regulate the activities of naturally-occuring autoreactive B lymphocytes (175). Although this theory and its numerous implications have been met with mixed response, the existence of anti-Id antibodies has been documented in numerous autoimmune diseases, including T1D (176–178). There is evidence that some healthy individuals harbor T1D associated AA against glutamic acid decarboxylase (GADA) as well as GADA-reactive anti-Id antibodies; the GADA are thought to be rendered ineffective by the neutralizing effects of the GADA-specific anti-Id antibodies which are present at significant levels in healthy individuals but depleted in newly-diagnosed T1D patients (178). Indeed, it has been proposed that a lack of anti-Id antibodies, rather than the presence of AA, may facilitate the progression of T1D in susceptible individuals (178) (**Figure 3**).

The generation of anti-Id antibodies directed against maternally derived antibodies has been described and explored in contexts such as allergy (179) and pathogen resistance (180) but very little in the context of T1D. It is known that anti-Id antibodies can be generated by the neonate in response to maternal IgG (180) or acquired from the mother (179). Several studies have demonstrated the ability of endogenous and maternally-delivered anti-Id antibodies to modify the B lymphocyte repertoire in neonatal mice, often resulting in suppression of the targeted B lymphocyte (181–183). Furthermore, there is evidence that T cell receptors (TCR) may also be a target for anti-Id antibody suppression in neonatal mice, resulting in early shaping of the T lymphocyte repertoire (184) (**Figure 3**). Given these findings, it is possible that maternally-induced idiotypic/anti-idiotypic interactions in the neonate play a role in neutralizing maternal AA activity and in modulating the neonatal lymphocyte repertoire. This could conceivably explain why maternally acquired AA in the circulation of neonates show no evidence of participating in or predisposing the child to autoimmune development despite being present in high concentrations. Furthermore, these anti-Id antibodies could leave a lasting impression on the developing lymphocyte repertoires such that naive lymphocytes with a propensity to recognize T1D associated autoantigen are suppressed or eliminated altogether. This complicated interplay between lymphocytes with idiotypically complimentary receptors could plausibly account for the reduced diabetes incidence reported in children from diabetic mothers; their immune systems may be primed for enhanced peripheral tolerance.

## 7 Conclusion

The under-investigated role of maternal influences in T1D is an intriguing area of study that so far has resulted in more questions than answers. Much of what has been learned about maternal microbiota and antibody transfer and their role in infant disease has been characterized outside of the context of T1D and thus requires a fair bit of speculation and hypothesizing with regards to their relevance to disease. It is broadly known that numerous maternal factors both *in utero* as well as postnatally have a strong and lasting effect on the health and immune development of the offspring. Mechanisms of these influences include early colonization, metabolite production and antigenic challenge by maternal microbes, establishing the infant gut microbiota and influencing the development of the gut environment and the immune system. Transfer of maternal antibodies against microbes and their metabolites further these aims, and allow for passive immune protection and establishment of gut homeostasis in infants.

The observations that hyperglycemia alters various aspects of lactation and humoral immunity suggest that the acquisition of maternally derived antibodies may be altered in the context of T1D. Moreover, the mounting evidence that maternally-derived T1D associated AA exhibit little to no pathogenicity in infants is an intriguing notion that demands further investigation. Furthermore, the fact that infants from T1D affected mothers exhibit reduced disease incidence despite evidence that maternal dysbiosis is reflected in neonatal biology suggests that the protective potential of maternal factors outweighs the pathogenic potential. We propose that it may be time to rethink dysbiosis and inflammation, as evidence for beneficial gut inflammation in the context of neonatal development are emerging and support a role of early exposure to diverse and/or immunogenic microorganisms in facilitating a more robust weaning reaction, ameliorating immune dysregulation, and reducing disease. Moreover, this theory has implications for studies that manipulate microbe sensing pathways. One must consider the possibility that the resulting phenotype from such studies may stem from a combination of altered microbial sensing in the offspring, as well as their early exposure to dysbiosis consequent to immunodeficiency. Maternal contributions from a dysbiotic dam during gestation and lactation may further confound the issue, urging caution in the interpretation of results from this type of study.

Finding a way to effectively halt autoimmune diabetes remains an unrealized goal. As we further our understanding of how maternally enacted mechanisms operate during the uniquely tolerogenic neonatal period, we open the door to the possibility of using maternal intervention strategies as a therapeutic means to prevent diseases such as T1D in the young.

## Author contributions

ES performed the literature search, wrote the manuscript and generated the figures. XC-C and ST edited and revised the manuscript. All authors contributed to the article and approved the submitted version.

## Glossary

**Table:**

T1D	type 1 diabetes
AA	autoantibodies
GI	gastrointestinal
EGF	epidermal growth factor
APC	antigen presenting cells
GAPs	goblet cell associated antigen passages
Tregs	CD4^+^ Foxp3^+^ regulatory T lymphocytes
SCFA	short chain fatty acids
IEC	intestinal epithelial cells
AhR	aryl hydrocarbon receptor
*Nfkb*	Nuclear factor kappa B gene
*Tlr2*	toll-like receptor 2 gene
*Tlr4*	toll-like receptor 4 gene
*Tslp*	Thymic stromal lymphopoietin gene
*Tnf*	tumor necrosis factor gene
*Ccl*2	chemokine (c-c motif) ligand 2; monocyte chemoattractant protein-1 gene
*Ccl3*	chemokine (c-c motif) ligand 3; macrophage inflammatory protein 1- alpha gene
*Cxcl1*	chemokine (c-x-c motif) ligand 1 gene
*Cxcl2*	chemokine (c-x-c motif) ligand 2 gene
*H2- Ea*	histocompatibility 2, class II antigen E alpha gene
MLN	mesenteric lymph node
*Reg3g*	regenerating islet-derived gene 3 gamma
*Reg3b*	regenerating islet-derived gene 3 beta
*Retnlb*	resistin like beta gene
IFNγ	interferon gamma
TNFα	tumor necrosis factor alpha
IL-10	interleukin 10
*Tap1*	Transporter associated with antigen processing 1 gene
*Tap2*	Transporter associated with antigen processing 2 gene
*Psmb8*	proteasome 20S subunit beta 8 gene
*Psmb9*	proteasome 20S subunit beta 9 gene
iTregs	inducible regulatory T lymphocytes
PP	Peyer’s Patches
NOD	non-obese diabetic mice
FcRn	neonatal Fc receptor
Ab-Ag	antibody-antigen complexes
OVA	ovalbumin
PPI-Fc	preproinsulin peptide fragment fused to Fc portion of IgG molecule
CVB1	Coxsackie virus B1
Tfh	T follicular helper cells
PLN	pancreatic lymph node
ILC3	type 3 innate lymphoid cells
IAA	insulin autoantibodies
ADCC	antibody dependent cellular cytotoxicity
ADCP	antibody dependent cellular phagocytosis
anti-Id	anti-idiotypic antibodies
GADA	glutamic acid decarboxylase autoantibodies
TCR	T cell receptor
PAMP	pathogen associated molecular patterns
GAD	glutamic acid decarboxylase
IA-2	tyrosine phosphatase-related insulinoma-associated 2 molecule

## References

[1] Bonifacio E Pflüger M Marienfeld S Winkler C Hummel M Ziegler A-G. Maternal type 1 diabetes reduces the risk of islet autoantibodies: Relationships with birthweight and maternal HbA(1c). Diabetologia (2008) 51:1245–52. doi: 10.1007/s00125-008-1022-z 18463843

[2] Warram JH Krolewski AS Gottlieb MS Kahn CR. Differences in risk of insulin-dependent diabetes in offspring of diabetic mothers and diabetic fathers. N Engl J Med (1984) 311:149–52. doi: 10.1056/NEJM198407193110304 6738600

[3] Familial risk of type I diabetes in European children. the eurodiab ace study group and the eurodiab ace substudy 2 study group. Diabetologia (1998) 41:1151–6. doi: 10.1007/s001250051044 9794100

[4] Harjutsalo V Reunanen A Tuomilehto J. Differential transmission of type 1 diabetes from diabetic fathers and mothers to their offspring. Diabetes (2006) 55:1517–24. doi: 10.2337/db05-1296 16644714

[5] Turtinen M Härkönen T Parkkola A Ilonen J Knip M Finnish Pediatric Diabetes Register. Characteristics of familial type 1 diabetes: Effects of the relationship to the affected family member on phenotype and genotype at diagnosis. Diabetologia (2019) 62:2025–39. doi: 10.1007/s00125-019-4952-8 PMC6805821 31346657

[6] Pacaud D Nucci AM Cuthbertson D Becker DJ Virtanen SM Ludvigsson J. Association between family history, early growth and the risk of beta cell autoimmunity in children at risk for type 1 diabetes. Diabetologia (2021) 64:119–28. doi: 10.1007/s00125-020-05287-1 PMC7716821 33026463

[7] Degnbol B Green A. Diabetes mellitus among first- and second-degree relatives of early onset diabetics. Ann Hum Genet (1978) 42:25–47. doi: 10.1111/j.1469-1809.1978.tb00929.x 686683

[8] Simpson NE. Heritabilities of liability to diabetes when sex and age at onset are considered. Ann Hum Genet (1969) 32:283–303. doi: 10.1111/j.1469-1809.1969.tb00077.x 5779422

[9] Wagener DK Sacks JM LaPorte RE Macgregor JM. The Pittsburgh study of insulin-dependent diabetes mellitus. Risk for diabetes among relatives of IDDM. Diabetes (1982) 31:136–44. doi: 10.2337/diab.31.2.136 6759229

[10] Dahlquist G Gustavsson KH Holmgren G Hägglöf B Larsson Y Nilsson KO. The incidence of diabetes mellitus in Swedish children 0-14 years of age. A prospective study 1977-1980. Acta Paediatr Scand (1982) 71:7–14. doi: 10.1111/j.1651-2227.1982.tb09364.x 6753469

[11] Waldhör T Schober E Rami B Tuomilehto J. The prevalence of IDDM in the first degree relatives of children newly diagnosed with IDDM in Austria–a population-based study. Austrian diabetes incidence study group. Exp Clin Endocrinol Diabetes (1999) 107:323–7. doi: 10.1055/s-0029-1212120 10482046

[12] Jamshidi P Hasanzadeh S Tahvildari A Farsi Y Arbabi M Mota JF. Is there any association between gut microbiota and type 1 diabetes? A systematic review. Gut Pathog (2019) 11:49. doi: 10.1186/s13099-019-0332-7 31636716 PMC6791003

[13] Han H Li Y Fang J Liu G Yin J Li T. Gut microbiota and type 1 diabetes. Int J Mol Sci (2018) 19:995. doi: 10.3390/ijms19040995 29584630 PMC5979537

[14] Dedrick S Sundaresh B Huang Q Brady C Yoo T Cronin C. The role of gut microbiota and environmental factors in type 1 diabetes pathogenesis. Front Endocrinol (Lausanne) (2020) 11:78. doi: 10.3389/fendo.2020.00078 32174888 PMC7057241

[15] Siljander H Honkanen J Knip M. Microbiome and type 1 diabetes. EBioMedicine (2019) 46:512–21. doi: 10.1016/j.ebiom.2019.06.031 PMC6710855 31257149

[16] Smith MJ Simmons KM Cambier JC. B cells in type 1 diabetes mellitus and diabetic kidney disease. Nat Rev Nephrol (2017) 13:712–20. doi: 10.1038/nrneph.2017.138 PMC6733025 29038537

[17] Simon AK Hollander GA McMichael A. Evolution of the immune system in humans from infancy to old age. Proc Biol Sci (2015) 282:20143085. doi: 10.1098/rspb.2014.3085 26702035 PMC4707740

[18] Gonzalez CA Gonzalez S. Fetal and neonatal allo-immune response. Transfus Apher Sci (2020) 59:102945. doi: 10.1016/j.transci.2020.102945 32958398

[19] Rackaityte E Halkias J. Mechanisms of fetal T cell tolerance and immune regulation. Front Immunol (2020) 11:588. doi: 10.3389/fimmu.2020.00588 32328065 PMC7160249

[20] Al Nabhani Z Dulauroy S Marques R Cousu C Al Bounny S Déjardin F. A weaning reaction to microbiota is required for resistance to immunopathologies in the adult. Immunity (2019) 50:1276–1288.e5. doi: 10.1016/j.immuni.2019.02.014 30902637

[21] El Aidy S van Baarlen P Derrien M Lindenbergh-Kortleve DJ Hooiveld G Levenez F. Temporal and spatial interplay of microbiota and intestinal mucosa drive establishment of immune homeostasis in conventionalized mice. Mucosal Immunol (2012) 5:567–79. doi: 10.1038/mi.2012.32 22617837

[22] Knoop KA Gustafsson JK McDonald KG Kulkarni D Coughlin PE McCrate S. Microbial antigen encounter during a pre-weaning interval is critical for tolerance to gut bacteria. Sci Immunol (2017) 2:eaao1314. doi: 10.1126/sciimmunol.aao1314 29246946 PMC5759965

[23] Craig ME Kim KW Isaacs SR Penno MA Hamilton-Williams EE Couper JJ. Early-life factors contributing to type 1 diabetes. Diabetologia (2019) 62:1823–34. doi: 10.1007/s00125-019-4942-x 31451871

[24] Tamburini S Shen N Wu HC Clemente JC. The microbiome in early life: Implications for health outcomes. Nat Med (2016) 22:713–22. doi: 10.1038/nm.4142 27387886

[25] Kennedy JL Haberling DL Huang CC Lessa FC Lucero DE Daskalakis DC. Infectious disease hospitalizations: United states, 2001 to 2014. Chest (2019) 156:255–68. doi: 10.1016/j.chest.2019.04.013 PMC6696939 31047954

[26] Cani PD. Human gut microbiome: Hopes, threats and promises. Gut (2018) 67:1716–25. doi: 10.1136/gutjnl-2018-316723 PMC6109275 29934437

[27] Wells JM Brummer RJ Derrien M MacDonald TT Troost F Cani PD. Homeostasis of the gut barrier and potential biomarkers. Am J Physiol Gastrointest Liver Physiol (2017) 312:G171–93. doi: 10.1152/ajpgi.00048.2015 PMC5440615 27908847

[28] Alexander M Turnbaugh PJ. Deconstructing mechanisms of diet-Microbiome-Immune interactions. Immunity (2020) 53:264–76. doi: 10.1016/j.immuni.2020.07.015 PMC7441819 32814025

[29] de Goffau MC Fuentes S van den Bogert B Honkanen H de Vos WM Welling GW. Aberrant gut microbiota composition at the onset of type 1 diabetes in young children. Diabetologia (2014) 57:1569–77. doi: 10.1007/s00125-014-3274-0 24930037

[30] Levy M Kolodziejczyk AA Thaiss CA Elinav E. Dysbiosis and the immune system. Nat Rev Immunol (2017) 17:219–32. doi: 10.1038/nri.2017.7 28260787

[31] Perez-Muñoz ME Arrieta M-C Ramer-Tait AE Walter J. A critical assessment of the “sterile womb” and “*in utero* colonization” hypotheses: Implications for research on the pioneer infant microbiome. Microbiome (2017) 5:48. doi: 10.1186/s40168-017-0268-4 28454555 PMC5410102

[32] Rutayisire E Huang K Liu Y Tao F. The mode of delivery affects the diversity and colonization pattern of the gut microbiota during the first year of infants’ life: A systematic review. BMC Gastroenterol (2016) 16:86. doi: 10.1186/s12876-016-0498-0 27475754 PMC4967522

[33] Azad MB Konya T Maughan H Guttman DS Field CJ Chari RS. Gut microbiota of healthy Canadian infants: Profiles by mode of delivery and infant diet at 4 months. CMAJ (2013) 185:385–94. doi: 10.1503/cmaj.121189 PMC3602254 23401405

[34] Tejesvi MV Nissi R Saravesi K Pirttilä AM Markkola A Talvensaari-Mattila A. Association of prevalent vaginal microbiome of mother with occurrence of type I diabetes in child. Sci Rep (2019) 9:959. doi: 10.1038/s41598-018-37467-w 30700742 PMC6353987

[35] Montoya-Williams D Lemas DJ Spiryda L Patel K Carney OO Neu J. The neonatal microbiome and its partial role in mediating the association between birth by cesarean section and adverse pediatric outcomes. Neonatology (2018) 114:103–11. doi: 10.1159/000487102 PMC6532636 29788027

[36] Daft JG Ptacek T Kumar R Morrow C Lorenz RG. Cross-fostering immediately after birth induces a permanent microbiota shift that is shaped by the nursing mother. Microbiome (2015) 3:17. doi: 10.1186/s40168-015-0080-y 25969735 PMC4427954

[37] Fehr K Moossavi S Sbihi H Boutin RCT Bode L Robertson B. Breastmilk feeding practices are associated with the Co-occurrence of bacteria in mothers’ milk and the infant gut: The CHILD cohort study. Cell Host Microbe (2020) 28:285–297.e4. doi: 10.1016/j.chom.2020.06.009 32652062

[38] Lackey KA Williams JE Meehan CL Zachek JA Benda ED Price WJ. What’s normal? Microbiomes in human milk and infant feces are related to each other but vary geographically: The INSPIRE study. Front Nutr (2019) 6:45. doi: 10.3389/fnut.2019.00045 31058158 PMC6479015

[39] Solís G de Los Reyes-Gavilan CG Fernández N Margolles A Gueimonde M. Establishment and development of lactic acid bacteria and bifidobacteria microbiota in breast-milk and the infant gut. Anaerobe (2010) 16:307–10. doi: 10.1016/j.anaerobe.2010.02.004 20176122

[40] Nolan LS Parks OB Good M. A review of the immunomodulating components of maternal breast milk and protection against necrotizing enterocolitis. Nutrients (2019) 12:E14. doi: 10.3390/nu12010014 PMC7019368 31861718

[41] Ballard O Morrow AL. Human milk composition: Nutrients and bioactive factors. Pediatr Clin North Am (2013) 60:49–74. doi: 10.1016/j.pcl.2012.10.002 23178060 PMC3586783

[42] Toscano M De Grandi R Grossi E Drago L. Role of the human breast milk-associated microbiota on the newborns’ immune system: A mini review. Front Microbiol (2017) 8:2100. doi: 10.3389/fmicb.2017.02100 29118752 PMC5661030

[43] Insel R Knip M. Prospects for primary prevention of type 1 diabetes by restoring a disappearing microbe. Pediatr Diabetes (2018) 19:1400–6. doi: 10.1111/pedi.12756 30136344

[44] Hidalgo-Cantabrana C Delgado S Ruiz L Ruas-Madiedo P Sánchez B Margolles A. Bifidobacteria and their health-promoting effects. Microbiol Spectr (2017) 5. doi: 10.1128/microbiolspec.BAD-0010-2016 PMC11687494 28643627

[45] Stewart CJ Ajami NJ O’Brien JL Hutchinson DS Smith DP Wong MC. Temporal development of the gut microbiome in early childhood from the TEDDY study. Nature (2018) 562:583–8. doi: 10.1038/s41586-018-0617-x PMC6415775 30356187

[46] Muñoz JAM Chenoll E Casinos B Bataller E Ramón D Genovés S. Novel probiotic bifidobacterium longum subsp. infantis CECT 7210 strain active against rotavirus infections. Appl Environ Microbiol (2011) 77:8775–83. doi: 10.1128/AEM.05548-11 PMC3233071 22003027

[47] Beghetti I Panizza D Lenzi J Gori D Martini S Corvaglia L. Probiotics for preventing necrotizing enterocolitis in preterm infants: A network meta-analysis. Nutrients (2021) 13:192. doi: 10.3390/nu13010192 33435456 PMC7827781

[48] Sonnenburg JL Chen CTL Gordon JI. Genomic and metabolic studies of the impact of probiotics on a model gut symbiont and host. PloS Biol (2006) 4:e413. doi: 10.1371/journal.pbio.0040413 17132046 PMC1661682

[49] Moens F Verce M De Vuyst L. Lactate- and acetate-based cross-feeding interactions between selected strains of lactobacilli, bifidobacteria and colon bacteria in the presence of inulin-type fructans. Int J Food Microbiol (2017) 241:225–36. doi: 10.1016/j.ijfoodmicro.2016.10.019 27810444

[50] Thananimit S Pahumunto N Teanpaisan R. Characterization of short chain fatty acids produced by selected potential probiotic lactobacillus strains. Biomolecules (2022) 12:1829. doi: 10.3390/biom12121829 36551257 PMC9775007

[51] Ziegler A-G Arnolds S Kölln A Achenbach P Berner R Bonifacio E. Supplementation with bifidobacterium longum subspecies infantis EVC001 for mitigation of type 1 diabetes autoimmunity: The GPPAD-SINT1A randomised controlled trial protocol. BMJ Open (2021) 11:e052449. doi: 10.1136/bmjopen-2021-052449 PMC8578987 34753762

[52] Fink LN Metzdorff SB Zeuthen LH Nellemann C Kristensen MB Licht TR. Establishment of tolerance to commensal bacteria requires a complex microbiota and is accompanied by decreased intestinal chemokine expression. Am J Physiol Gastrointest Liver Physiol (2012) 302:G55–65. doi: 10.1152/ajpgi.00428.2010 21960522

[53] Ashraf R Shah NP. Immune system stimulation by probiotic microorganisms. Crit Rev Food Sci Nutr (2014) 54:938–56. doi: 10.1080/10408398.2011.619671 24499072

[54] Galdeano CM de LeBlanc AdeM Carmuega E Weill R Perdigón G. Mechanisms involved in the immunostimulation by probiotic fermented milk. J Dairy Res (2009) 76:446–54. doi: 10.1017/S0022029909990021 19638260

[55] de Moreno de LeBlanc A Dogi CA Galdeano CM Carmuega E Weill R Perdigón G. Effect of the administration of a fermented milk containing lactobacillus casei DN-114001 on intestinal microbiota and gut associated immune cells of nursing mice and after weaning until immune maturity. BMC Immunol (2008) 9:27. doi: 10.1186/1471-2172-9-27 18554392 PMC2459154

[56] Li H Limenitakis JP Greiff V Yilmaz B Schären O Urbaniak C. Mucosal or systemic microbiota exposures shape the b cell repertoire. Nature (2020) 584:274–8. doi: 10.1038/s41586-020-2564-6 32760003

[57] Zegarra-Ruiz DF Kim DV Norwood K Kim M Wu W-JH Saldana-Morales FB. Thymic development of gut-microbiota-specific T cells. Nature (2021) 594:413–7. doi: 10.1038/s41586-021-03531-1 PMC8323488 33981034

[58] Cebula A Seweryn M Rempala GA Pabla SS McIndoe RA Denning TL. Thymus-derived regulatory T cells contribute to tolerance to commensal microbiota. Nature (2013) 497:258–62. doi: 10.1038/nature12079 PMC3711137 23624374

[59] Wood H Acharjee A Pearce H Quraishi MN Powell R Rossiter A. Breastfeeding promotes early neonatal regulatory T-cell expansion and immune tolerance of non-inherited maternal antigens. Allergy (2021) 76:2447–60. doi: 10.1111/all.14736 33432577

[60] Yu JC Khodadadi H Malik A Davidson B Salles É da SL Bhatia J. Innate immunity of neonates and infants. Front Immunol (2018) 9:1759. doi: 10.3389/fimmu.2018.01759 30105028 PMC6077196

[61] Vergani S Muleta KG Da Silva C Doyle A Kristiansen TA Sodini S. A self-sustaining layer of early-life-origin b cells drives steady-state IgA responses in the adult gut. Immunity (2022) 55:1829–1842.e6. doi: 10.1016/j.immuni.2022.08.018 36115337

[62] Sefik E Geva-Zatorsky N Oh S Konnikova L Zemmour D McGuire AM. Individual intestinal symbionts induce a distinct population of RORγ+ regulatory T cells. Science (2015) 349:993–7. doi: 10.1126/science.aaa9420 PMC4700932 26272906

[63] Zhang X Borbet TC Fallegger A Wipperman MF Blaser MJ Müller A. An antibiotic-impacted microbiota compromises the development of colonic regulatory T cells and predisposes to dysregulated immune responses. mBio (2021) 12:e03335–20. doi: 10.1128/mBio.03335-20 PMC7858066 33531385

[64] Needell JC Ir D Robertson CE Kroehl ME Frank DN Zipris D. Maternal treatment with short-chain fatty acids modulates the intestinal microbiota and immunity and ameliorates type 1 diabetes in the offspring. PloS One (2017) 12:e0183786. doi: 10.1371/journal.pone.0183786 28886045 PMC5590848

[65] Jia L Cao M Chen H Zhang M Dong X Ren Z. Butyrate ameliorates antibiotic-driven type 1 diabetes in the female offspring of nonobese diabetic mice. J Agric Food Chem (2020) 68:3112–20. doi: 10.1021/acs.jafc.9b07701 32046486

[66] Harbison JE Roth-Schulze AJ Giles LC Tran CD Ngui KM Penno MA. Gut microbiome dysbiosis and increased intestinal permeability in children with islet autoimmunity and type 1 diabetes: A prospective cohort study. Pediatr Diabetes (2019) 20:574–83. doi: 10.1111/pedi.12865 31081243

[67] Kostic AD Gevers D Siljander H Vatanen T Hyötyläinen T Hämäläinen A-M. The dynamics of the human infant gut microbiome in development and in progression toward type 1 diabetes. Cell Host Microbe (2015) 17:260–73. doi: 10.1016/j.chom.2015.01.001 PMC4689191 25662751

[68] Maffeis C Martina A Corradi M Quarella S Nori N Torriani S. Association between intestinal permeability and faecal microbiota composition in Italian children with beta cell autoimmunity at risk for type 1 diabetes. Diabetes Metab Res Rev (2016) 32:700–9. doi: 10.1002/dmrr.2790 26891226

[69] de Goffau MC Luopajärvi K Knip M Ilonen J Ruohtula T Härkönen T. Fecal microbiota composition differs between children with β-cell autoimmunity and those without. Diabetes (2013) 62:1238–44. doi: 10.2337/db12-0526 PMC3609581 23274889

[70] Brown CT Davis-Richardson AG Giongo A Gano KA Crabb DB Mukherjee N. Gut microbiome metagenomics analysis suggests a functional model for the development of autoimmunity for type 1 diabetes. PloS One (2011) 6:e25792. doi: 10.1371/journal.pone.0025792 22043294 PMC3197175

[71] Zhao G Vatanen T Droit L Park A Kostic AD Poon TW. Intestinal virome changes precede autoimmunity in type I diabetes-susceptible children. Proc Natl Acad Sci U.S.A. (2017) 114:E6166–75. doi: 10.1073/pnas.1706359114 PMC5544325 28696303

[72] van Heck JIP Gacesa R Stienstra R Fu J Zhernakova A Harmsen HJM. The gut microbiome composition is altered in long-standing type 1 diabetes and associates with glycemic control and disease-related complications. Diabetes Care (2022) 45:2084–94. doi: 10.2337/dc21-2225 35766965

[73] Vatanen T Franzosa EA Schwager R Tripathi S Arthur TD Vehik K. The human gut microbiome in early-onset type 1 diabetes from the TEDDY study. Nature (2018) 562:589–94. doi: 10.1038/s41586-018-0620-2 PMC6296767 30356183

[74] Sorini C Cosorich I Lo Conte M De Giorgi L Facciotti F Lucianò R. Loss of gut barrier integrity triggers activation of islet-reactive T cells and autoimmune diabetes. Proc Natl Acad Sci USA (2019) 116:15140–9. doi: 10.1073/pnas.1814558116 PMC6660755 31182588

[75] Costa FRC Françozo MCS de Oliveira GG Ignacio A Castoldi A Zamboni DS. Gut microbiota translocation to the pancreatic lymph nodes triggers NOD2 activation and contributes to T1D onset. J Exp Med (2016) 213:1223–39. doi: 10.1084/jem.20150744 PMC4925011 27325889

[76] Vaarala O Atkinson MA Neu J. The “perfect storm” for type 1 diabetes: The complex interplay between intestinal microbiota, gut permeability, and mucosal immunity. Diabetes (2008) 57:2555–62. doi: 10.2337/db08-0331 PMC2551660 18820210

[77] Mønsted MØ Falck ND Pedersen K Buschard K Holm LJ Haupt-Jorgensen M. Intestinal permeability in type 1 diabetes: An updated comprehensive overview. J Autoimmun (2021) 122:102674. doi: 10.1016/j.jaut.2021.102674 34182210

[78] Tai N Wong FS Wen L. The role of the innate immune system in destruction of pancreatic beta cells in NOD mice and humans with type I diabetes. J Autoimmun (2016) 71:26–34. doi: 10.1016/j.jaut.2016.03.006 27021275 PMC4903935

[79] Wen L Ley RE Volchkov PY Stranges PB Avanesyan L Stonebraker AC. Innate immunity and intestinal microbiota in the development of type 1 diabetes. Nature (2008) 455:1109–13. doi: 10.1038/nature07336 PMC2574766 18806780

[80] Brown K Godovannyi A Ma C Zhang Y Ahmadi-Vand Z Dai C. Prolonged antibiotic treatment induces a diabetogenic intestinal microbiome that accelerates diabetes in NOD mice. ISME J (2016) 10:321–32. doi: 10.1038/ismej.2015.114 PMC4737925 26274050

[81] Valladares R Sankar D Li N Williams E Lai K-K Abdelgeliel AS. Lactobacillus johnsonii N6.2 mitigates the development of type 1 diabetes in BB-DP rats. PloS One (2010) 5:e10507. doi: 10.1371/journal.pone.0010507 20463897 PMC2865539

[82] Hänninen A Toivonen R Pöysti S Belzer C Plovier H Ouwerkerk JP. Akkermansia muciniphila induces gut microbiota remodelling and controls islet autoimmunity in NOD mice. Gut (2018) 67:1445–53. doi: 10.1136/gutjnl-2017-314508 29269438

[83] Markle JGM Frank DN Mortin-Toth S Robertson CE Feazel LM Rolle-Kampczyk U. Sex differences in the gut microbiome drive hormone-dependent regulation of autoimmunity. Science (2013) 339:1084–8. doi: 10.1126/science.1233521 23328391

[84] de Groot P Nikolic T Pellegrini S Sordi V Imangaliyev S Rampanelli E. Faecal microbiota transplantation halts progression of human new-onset type 1 diabetes in a randomised controlled trial. Gut (2021) 70:92–105. doi: 10.1136/gutjnl-2020-322630 33106354 PMC7788262

[85] Allen DW Kim KW Rawlinson WD Craig ME. Maternal virus infections in pregnancy and type 1 diabetes in their offspring: Systematic review and meta-analysis of observational studies. Rev Med Virol (2018) 28:e1974. doi: 10.1002/rmv.1974 29569297

[86] Quinn LM Wong FS Narendran P. Environmental determinants of type 1 diabetes: From association to proving causality. Front Immunol (2021) 12:737964. doi: 10.3389/fimmu.2021.737964 34659229 PMC8518604

[87] Yeung W-CG Rawlinson WD Craig ME. Enterovirus infection and type 1 diabetes mellitus: Systematic review and meta-analysis of observational molecular studies. BMJ (2011) 342:d35. doi: 10.1136/bmj.d35 21292721 PMC3033438

[88] Lin H-C Wang C-H Tsai F-J Hwang K-P Chen W Lin C-C. Enterovirus infection is associated with an increased risk of childhood type 1 diabetes in Taiwan: A nationwide population-based cohort study. Diabetologia (2015) 58:79–86. doi: 10.1007/s00125-014-3400-z 25335440

[89] Kordonouri O Cuthbertson D Belteky M Aschemeier-Fuchs B White NH Cummings E. Infections in the first year of life and development of beta cell autoimmunity and clinical type 1 diabetes in high-risk individuals: The TRIGR cohort. Diabetologia (2022) 65:2098–107. doi: 10.1007/s00125-022-05786-3 PMC9630400 36083343

[90] Ponsonby A-L Pezic A Cochrane J Cameron FJ Pascoe M Kemp A. Infant anthropometry, early life infection, and subsequent risk of type 1 diabetes mellitus: A prospective birth cohort study. Pediatr Diabetes (2011) 12:313–21. doi: 10.1111/j.1399-5448.2010.00693.x 21615650

[91] Richer MJ Horwitz MS. Coxsackievirus infection as an environmental factor in the etiology of type 1 diabetes. Autoimmun Rev (2009) 8:611–5. doi: 10.1016/j.autrev.2009.02.006 19393207

[92] Vatanen T Kostic AD d’Hennezel E Siljander H Franzosa EA Yassour M. Variation in microbiome LPS immunogenicity contributes to autoimmunity in humans. Cell (2016) 165:842–53. doi: 10.1016/j.cell.2016.04.007 PMC4950857 27133167

[93] Kondrashova A Reunanen A Romanov A Karvonen A Viskari H Vesikari T. A six-fold gradient in the incidence of type 1 diabetes at the eastern border of Finland. Ann Med (2005) 37:67–72. doi: 10.1080/07853890410018952 15902849

[94] Hu J Nomura Y Bashir A Fernandez-Hernandez H Itzkowitz S Pei Z. Diversified microbiota of meconium is affected by maternal diabetes status. PloS One (2013) 8:e78257. doi: 10.1371/journal.pone.0078257 24223144 PMC3819383

[95] Wang J Zheng J Shi W Du N Xu X Zhang Y. Dysbiosis of maternal and neonatal microbiota associated with gestational diabetes mellitus. Gut (2018) 67:1614–25. doi: 10.1136/gutjnl-2018-315988 PMC6109274 29760169

[96] Su M Nie Y Shao R Duan S Jiang Y Wang M. Diversified gut microbiota in newborns of mothers with gestational diabetes mellitus. PloS One (2018) 13:e0205695. doi: 10.1371/journal.pone.0205695 30332459 PMC6192631

[97] Zhao C Ge J Li X Jiao R Li Y Quan H. Integrated metabolome analysis reveals novel connections between maternal fecal metabolome and the neonatal blood metabolome in women with gestational diabetes mellitus. Sci Rep (2020) 10:3660. doi: 10.1038/s41598-020-60540-2 32107447 PMC7046769

[98] Ruotsalainen AL Tejesvi MV Vänni P Suokas M Tossavainen P Pirttilä AM. Child type 1 diabetes associated with mother vaginal bacteriome and mycobiome. Med Microbiol Immunol (2022) 211:185–94. doi: 10.1007/s00430-022-00741-w PMC9304052 35701558

[99] Honkanen J Vuorela A Muthas D Orivuori L Luopajärvi K Tejesvi MVG. Fungal dysbiosis and intestinal inflammation in children with beta-cell autoimmunity. Front Immunol (2020) 11:468. doi: 10.3389/fimmu.2020.00468 32265922 PMC7103650

[100] Stinson LF Sindi ASM Cheema AS Lai CT Mühlhäusler BS Wlodek ME. The human milk microbiome: Who, what, when, where, why, and how? Nutr Rev (2021) 79:529–43. doi: 10.1093/nutrit/nuaa029 32443154

[101] Gámez-Valdez JS García-Mazcorro JF Montoya-Rincón AH Rodríguez-Reyes DL Jiménez-Blanco G Rodríguez MTA. Differential analysis of the bacterial community in colostrum samples from women with gestational diabetes mellitus and obesity. Sci Rep (2021) 11:24373. doi: 10.1038/s41598-021-03779-7 34934118 PMC8692321

[102] Nyangahu DD Lennard KS Brown BP Darby MG Wendoh JM Havyarimana E. Disruption of maternal gut microbiota during gestation alters offspring microbiota and immunity. Microbiome (2018) 6:124. doi: 10.1186/s40168-018-0511-7 29981583 PMC6035804

[103] Usami K Niimi K Matsuo A Suyama Y Sakai Y Sato S. The gut microbiota induces peyer’s-patch-dependent secretion of maternal IgA into milk. Cell Rep (2021) 36:109655. doi: 10.1016/j.celrep.2021.109655 34496253

[104] Patelarou E Girvalaki C Brokalaki H Patelarou A Androulaki Z Vardavas C. Current evidence on the associations of breastfeeding, infant formula, and cow’s milk introduction with type 1 diabetes mellitus: A systematic review. Nutr Rev (2012) 70:509–19. doi: 10.1111/j.1753-4887.2012.00513.x 22946851

[105] Lampousi A-M Carlsson S Löfvenborg JE. Dietary factors and risk of islet autoimmunity and type 1 diabetes: A systematic review and meta-analysis. EBioMedicine (2021) 72:103633. doi: 10.1016/j.ebiom.2021.103633 34656932 PMC8523874

[106] Miranda MCG Oliveira RP Torres L Aguiar SLF Pinheiro-Rosa N Lemos L. Frontline science: Abnormalities in the gut mucosa of non-obese diabetic mice precede the onset of type 1 diabetes. J Leukoc Biol (2019) 106:513–29. doi: 10.1002/JLB.3HI0119-024RR 31313381

[107] Johansson MEV. Mucus layers in inflammatory bowel disease. Inflammation Bowel Dis (2014) 20:2124–31. doi: 10.1097/MIB.0000000000000117 25025717

[108] Maruyama K Hida M Kohgo T Fukunaga Y. Changes in salivary and fecal secretory IgA in infants under different feeding regimens. Pediatr Int (2009) 51:342–5. doi: 10.1111/j.1442-200X.2008.02748.x 19400812

[109] Bridgman SL Konya T Azad MB Sears MR Becker AB Turvey SE. Infant gut immunity: A preliminary study of IgA associations with breastfeeding. J Dev Orig Health Dis (2016) 7:68–72. doi: 10.1017/S2040174415007862 26690933

[110] Zheng W Zhao W Wu M Song X Caro F Sun X. Microbiota-targeted maternal antibodies protect neonates from enteric infection. Nature (2020) 577:543–8. doi: 10.1038/s41586-019-1898-4 PMC7362890 31915378

[111] Caballero-Flores G Sakamoto K Zeng MY Wang Y Hakim J Matus-Acuña V. Maternal immunization confers protection to the offspring against an attaching and effacing pathogen through delivery of IgG in breast milk. Cell Host Microbe (2019) 25:313–323.e4. doi: 10.1016/j.chom.2018.12.015 30686564 PMC6375740

[112] Rogier EW Frantz AL Bruno MEC Kaetzel CS. Secretory IgA is concentrated in the outer layer of colonic mucus along with gut bacteria. Pathogens (2014) 3:390–403. doi: 10.3390/pathogens3020390 25437806 PMC4243452

[113] Torow N Yu K Hassani K Freitag J Schulz O Basic M. Active suppression of intestinal CD4(+)TCRαβ(+) T-lymphocyte maturation during the postnatal period. Nat Commun (2015) 6:7725. doi: 10.1038/ncomms8725 26195040 PMC4518322

[114] França EL Morceli G Fagundes DLG Rudge MVC Calderon IDMP Honorio-França AC. Secretory IgA-fcα receptor interaction modulating phagocytosis and microbicidal activity by phagocytes in human colostrum of diabetics. APMIS (2011) 119:710–9. doi: 10.1111/j.1600-0463.2011.02789.x 21917008

[115] Monteiro RC Van De Winkel JGJ. IgA fc receptors. Annu Rev Immunol (2003) 21:177–204. doi: 10.1146/annurev.immunol.21.120601.141011 12524384

[116] Hartmann PE Cregan MD Mitoulas LR. Maternal modulation of specific and non-specific immune components of colostrum and mature milk. Adv Nutr Res (2001) 10:365–87. doi: 10.1007/978-1-4615-0661-4_18 11795051

[117] França EL Calderon I de MP Vieira EL Morceli G Honorio-França AC. Transfer of maternal immunity to newborns of diabetic mothers. Clin Dev Immunol (2012) 2012:928187. doi: 10.1155/2012/928187 22991568 PMC3444004

[118] Peila C Gazzolo D Bertino E Cresi F Coscia A. Influence of diabetes during pregnancy on human milk composition. Nutrients (2020) 12:E185. doi: 10.3390/nu12010185 PMC7019231 31936574

[119] Smilowitz JT Totten SM Huang J Grapov D Durham HA Lammi-Keefe CJ. Human milk secretory immunoglobulin a and lactoferrin n-glycans are altered in women with gestational diabetes mellitus. J Nutr (2013) 143:1906–12. doi: 10.3945/jn.113.180695 PMC3827637 24047700

[120] Leach L Eaton BM Firth JA Contractor SF. Uptake and intracellular routing of peroxidase-conjugated immunoglobulin-G by the perfused human placenta. Cell Tissue Res (1990) 261:383–8. doi: 10.1007/BF00318681 2401009

[121] Leach L Eaton BM Firth JA Contractor SF. Immunocytochemical and labelled tracer approaches to uptake and intracellular routing of immunoglobulin-G (IgG) in the human placenta. Histochem J (1991) 23:444–9. doi: 10.1007/BF01041374 1743992

[122] Mimoun A Delignat S Peyron I Daventure V Lecerf M Dimitrov JD. Relevance of the materno-fetal interface for the induction of antigen-specific immune tolerance. Front Immunol (2020) 11:810. doi: 10.3389/fimmu.2020.00810 32477339 PMC7240014

[123] Mikulska JE Pablo L Canel J Simister NE. Cloning and analysis of the gene encoding the human neonatal fc receptor. Eur J Immunogenet (2000) 27:231–40. doi: 10.1046/j.1365-2370.2000.00225.x 10998088

[124] Tiwari B Junghans RP. Functional analysis of the mouse fcgrt 5’ proximal promoter. Biochim Biophys Acta (2005) 1681:88–98. doi: 10.1016/j.bbaexp.2004.10.002 15627500

[125] Ahouse JJ Hagerman CL Mittal P Gilbert DJ Copeland NG Jenkins NA. Mouse MHC class I-like fc receptor encoded outside the MHC. J Immunol (1993) 151:6076–88. doi: 10.4049/jimmunol.151.11.6076 7504013

[126] Latvala S Jacobsen B Otteneder MB Herrmann A Kronenberg S. Distribution of FcRn across species and tissues. J Histochem Cytochem (2017) 65:321–33. doi: 10.1369/0022155417705095 PMC5625855 28402755

[127] Appleby P Catty D. Transmission of immunoglobulin to foetal and neonatal mice. J Reprod Immunol (1983) 5:203–13. doi: 10.1016/0165-0378(83)90236-x 6620251

[128] Montoyo HP Vaccaro C Hafner M Ober RJ Mueller W Ward ES. Conditional deletion of the MHC class I-related receptor FcRn reveals the sites of IgG homeostasis in mice. Proc Natl Acad Sci U.S.A. (2009) 106:2788–93. doi: 10.1073/pnas.0810796106 PMC2650344 19188594

[129] Jones EA Waldmann TA. The mechanism of intestinal uptake and transcellular transport of IgG in the neonatal rat. J Clin Invest (1972) 51:2916–27. doi: 10.1172/JCI107116 PMC292442 5080417

[130] Balfour AH Jones EA. The binding of plasma proteins to human placental cell membranes. Clin Sci Mol Med (1977) 52:383–94. doi: 10.1042/cs0520383 862334

[131] Kristoffersen EK Matre R. Co-Localization of the neonatal fc gamma receptor and IgG in human placental term syncytiotrophoblasts. Eur J Immunol (1996) 26:1668–71. doi: 10.1002/eji.1830260741 8766579

[132] Leach JL Sedmak DD Osborne JM Rahill B Lairmore MD Anderson CL. Isolation from human placenta of the IgG transporter, FcRn, and localization to the syncytiotrophoblast: Implications for maternal-fetal antibody transport. J Immunol (1996) 157:3317–22. doi: 10.4049/jimmunol.157.8.3317 8871627

[133] Antohe F Rădulescu L Gafencu A Gheţie V Simionescu M. Expression of functionally active FcRn and the differentiated bidirectional transport of IgG in human placental endothelial cells. Hum Immunol (2001) 62:93–105. doi: 10.1016/s0198-8859(00)00244-5 11182218

[134] Kiskova T Mytsko Y Schepelmann M Helmer H Fuchs R Miedl H. Expression of the neonatal fc-receptor in placental-fetal endothelium and in cells of the placental immune system. Placenta (2019) 78:36–43. doi: 10.1016/j.placenta.2019.02.012 30955709

[135] Simister NE Story C Chen H Hunt H. An IgG-transporting fc receptor expressed in the syncytiotrophoblast of human placenta. Eur J Immunol (1996) 26:1527–31. doi: 10.1002/eji.1830260718 8766556

[136] Kohler PF Farr RS. Elevation of cord over maternal IgG immunoglobulin: Evidence for an active placental IgG transport. Nature (1966) 210:1070–1. doi: 10.1038/2101070a0 5950290

[137] Malek A Sager R Schneider H. Maternal-fetal transport of immunoglobulin G and its subclasses during the third trimester of human pregnancy. Am J Reprod Immunol (1994) 32:8–14. doi: 10.1111/j.1600-0897.1994.tb00873.x 7945815

[138] Malek A Sager R Kuhn P Nicolaides KH Schneider H. Evolution of maternofetal transport of immunoglobulins during human pregnancy. Am J Reprod Immunol (1996) 36:248–55. doi: 10.1111/j.1600-0897.1996.tb00172.x 8955500

[139] Zhu X Meng G Dickinson BL Li X Mizoguchi E Miao L. MHC class I-related neonatal fc receptor for IgG is functionally expressed in monocytes, intestinal macrophages, and dendritic cells. J Immunol (2001) 166:3266–76. doi: 10.4049/jimmunol.166.5.3266 PMC2827247 11207281

[140] Vidarsson G Stemerding AM Stapleton NM Spliethoff SE Janssen H Rebers FE. FcRn: An IgG receptor on phagocytes with a novel role in phagocytosis. Blood (2006) 108:3573–9. doi: 10.1182/blood-2006-05-024539 16849638

[141] Bentley JP Simpson JM Bowen JR Morris JM Roberts CL Nassar N. Gestational age, mode of birth and breastmilk feeding all influence acute early childhood gastroenteritis: A record-linkage cohort study. BMC Pediatr (2016) 16:55. doi: 10.1186/s12887-016-0591-0 27122131 PMC4847338

[142] Le Doare K Bellis K Faal A Birt J Munblit D Humphries H. SIgA, TGF-β1, IL-10, and TNFα in colostrum are associated with infant group b streptococcus colonization. Front Immunol (2017) 8:1269. doi: 10.3389/fimmu.2017.01269 29109718 PMC5660603

[143] Nicoara C Zäch K Trachsel D Germann D Matter L. Decay of passively acquired maternal antibodies against measles, mumps, and rubella viruses. Clin Diagn Lab Immunol (1999) 6:868–71. doi: 10.1128/CDLI.6.6.868-871.1999 PMC95790 10548578

[144] Benowitz I Esposito DB Gracey KD Shapiro ED Vázquez M. Influenza vaccine given to pregnant women reduces hospitalization due to influenza in their infants. Clin Infect Dis (2010) 51:1355–61. doi: 10.1086/657309 PMC3106242 21058908

[145] Ward ES Zhou J Ghetie V Ober RJ. Evidence to support the cellular mechanism involved in serum IgG homeostasis in humans. Int Immunol (2003) 15:187–95. doi: 10.1093/intimm/dxg018 12578848

[146] Giacoia GP. Transplacentally transmitted autoimmune disorders of the fetus and newborn: Pathogenic considerations. South Med J (1992) 85:139–45. doi: 10.1097/00007611-199202000-00006 1738879

[147] de Souza EG Hara CCP Fagundes DLG de Queiroz AA Morceli G Calderon IMP. Maternal-foetal diabetes modifies neonatal fc receptor expression on human leucocytes. Scand J Immunol (2016) 84:237–44. doi: 10.1111/sji.12466 27469170

[148] Koch MA Reiner GL Lugo KA Kreuk LSM Stanbery AG Ansaldo E. Maternal IgG and IgA antibodies dampen mucosal T helper cell responses in early life. Cell (2016) 165:827–41. doi: 10.1016/j.cell.2016.04.055 PMC4866587 27153495

[149] Polte T Hansen G. Maternal tolerance achieved during pregnancy is transferred to the offspring *via* breast milk and persistently protects the offspring from allergic asthma. Clin Exp Allergy (2008) 38:1950–8. doi: 10.1111/j.1365-2222.2008.03096.x 18778271

[150] Mosconi E Rekima A Seitz-Polski B Kanda A Fleury S Tissandie E. Breast milk immune complexes are potent inducers of oral tolerance in neonates and prevent asthma development. Mucosal Immunol (2010) 3:461–74. doi: 10.1038/mi.2010.23 20485331

[151] Ohsaki A Venturelli N Buccigrosso TM Osganian SK Lee J Blumberg RS. Maternal IgG immune complexes induce food allergen-specific tolerance in offspring. J Exp Med (2018) 215:91–113. doi: 10.1084/jem.20171163 29158374 PMC5748859

[152] Culina S Gupta N Boisgard R Afonso G Gagnerault M-C Dimitrov J. Materno-fetal transfer of preproinsulin through the neonatal fc receptor prevents autoimmune diabetes. Diabetes (2015) 64:3532–42. doi: 10.2337/db15-0024 25918233

[153] Corcos N Culina S Deligne C Lavaud C You S Mallone R. Oral fc-coupled preproinsulin achieves systemic and thymic delivery through the neonatal fc receptor and partially delays autoimmune diabetes. Front Immunol (2021) 12:616215. doi: 10.3389/fimmu.2021.616215 34447366 PMC8382691

[154] Lind A Lynch KF Lundgren M Lernmark Å Almgren P Ramelius A. First trimester enterovirus IgM and beta cell autoantibodies in mothers to children affected by type 1 diabetes autoimmunity before 7 years of age. J Reprod Immunol (2018) 127:1–6. doi: 10.1016/j.jri.2018.02.004 29550618

[155] Viskari HR Koskela P Lönnrot M Luonuansuu S Reunanen A Baer M. Can enterovirus infections explain the increasing incidence of type 1 diabetes? Diabetes Care (2000) 23:414–6. doi: 10.2337/diacare.23.3.414 10868874

[156] Viskari H Ludvigsson J Uibo R Salur L Marciulionyte D Hermann R. Relationship between the incidence of type 1 diabetes and maternal enterovirus antibodies: Time trends and geographical variation. Diabetologia (2005) 48:1280–7. doi: 10.1007/s00125-005-1780-9 15902401

[157] Horwitz MS Bradley LM Harbertson J Krahl T Lee J Sarvetnick N. Diabetes induced by coxsackie virus: Initiation by bystander damage and not molecular mimicry. Nat Med (1998) 4:781–5. doi: 10.1038/nm0798-781 9662368

[158] Larsson PG Lakshmikanth T Svedin E King C Flodström-Tullberg M. Previous maternal infection protects offspring from enterovirus infection and prevents experimental diabetes development in mice. Diabetologia (2013) 56:867–74. doi: 10.1007/s00125-013-2834-z 23344730

[159] Laitinen OH Honkanen H Pakkanen O Oikarinen S Hankaniemi MM Huhtala H. Coxsackievirus B1 is associated with induction of β-cell autoimmunity that portends type 1 diabetes. Diabetes (2014) 63:446–55. doi: 10.2337/db13-0619 23974921

[160] Gomez de Agüero M Ganal-Vonarburg SC Fuhrer T Rupp S Uchimura Y Li H. The maternal microbiota drives early postnatal innate immune development. Science (2016) 351:1296–302. doi: 10.1126/science.aad2571 26989247

[161] Waltuck J Buyon JP. Autoantibody-associated congenital heart block: Outcome in mothers and children. Ann Intern Med (1994) 120:544–51. doi: 10.7326/0003-4819-120-7-199404010-00003 8116991

[162] Greeley SAW Katsumata M Yu L Eisenbarth GS Moore DJ Goodarzi H. Elimination of maternally transmitted autoantibodies prevents diabetes in nonobese diabetic mice. Nat Med (2002) 8:399–402. doi: 10.1038/nm0402-399 11927947

[163] Koczwara K Ziegler A-G Bonifacio E. Maternal immunity to insulin does not affect diabetes risk in progeny of non obese diabetic mice. Clin Exp Immunol (2004) 136:56–9. doi: 10.1111/j.1365-2249.2004.02406.x PMC1809008 15030514

[164] Colman PG Steele C Couper JJ Beresford SJ Powell T Kewming K. Islet autoimmunity in infants with a type I diabetic relative is common but is frequently restricted to one autoantibody. Diabetologia (2000) 43:203–9. doi: 10.1007/s001250050030 10753042

[165] Naserke HE Bonifacio E Ziegler AG. Prevalence, characteristics and diabetes risk associated with transient maternally acquired islet antibodies and persistent islet antibodies in offspring of parents with type 1 diabetes. J Clin Endocrinol Metab (2001) 86:4826–33. doi: 10.1210/jcem.86.10.7931 11600549

[166] Stanley HM Norris JM Barriga K Hoffman M Yu L Miao D. Is presence of islet autoantibodies at birth associated with development of persistent islet autoimmunity? The diabetes autoimmunity study in the young (DAISY). Diabetes Care (2004) 27:497–502. doi: 10.2337/diacare.27.2.497 14747235

[167] Hämäläinen AM Savola K Kulmala PK Koskela P Akerblom HK Knip M. Disease-associated autoantibodies during pregnancy and at birth in families affected by type 1 diabetes. Clin Exp Immunol (2001) 126:230–5. doi: 10.1046/j.1365-2249.2001.01676.x PMC1906201 11703365

[168] Colten HR Goldberger G. Ontogeny of serum complement proteins. Pediatrics (1979) 64:775–80. doi: 10.1542/peds.64.5.775 503698

[169] Ballow M Fang F Good RA Day NK. Developmental aspects of complement components in the newborn. the presence of complement components and C3 proactivator (properdin factor b) in human colostrum. Clin Exp Immunol (1974) 18:257–66. PMC1537884 4478765

[170] Roll U Christie MR Füchtenbusch M Payton MA Hawkes CJ Ziegler AG. Perinatal autoimmunity in offspring of diabetic parents. the German multicenter BABY-DIAB study: Detection of humoral immune responses to islet antigens in early childhood. Diabetes (1996) 45:967–73. doi: 10.2337/diab.45.7.967 8666150

[171] CDC. Type 1 diabetes and pregnancy. centers for disease control and prevention (2021). Available at: https://www.cdc.gov/diabetes/library/features/type-1-and-pregnancy.html (Accessed September 12, 2022).

[172] Diabetes and breastfeeding | ADA . Available at: https://diabetes.org/diabetes/gestational-diabetes/diabetes-breastfeeding (Accessed September 12, 2022).

[173] Breastfeeding and diabetes - type 1, type 2 and gestational diabetes | Australian breastfeeding association. Available at: https://www.breastfeeding.asn.au/resources/breastfeeding-and-diabetes-type-1-type-2-and-gestational-diabetes (Accessed September 12, 2022).

[174] Jerne NK. Towards a network theory of the immune system. Ann Immunol (Paris) (1974) 125C:373–89. 4142565

[175] Hampe CS. Protective role of anti-idiotypic antibodies in autoimmunity–lessons for type 1 diabetes. Autoimmunity (2012) 45:320–31. doi: 10.3109/08916934.2012.659299 PMC5319419 22288464

[176] Casiglia D Giardina E Triolo G. IgG auto-anti-idiotype antibodies against antibody to insulin in insulin-dependent (type 1) diabetes mellitus. detection by capture enzyme linked immunosorbent assay (ELISA) and relationship with anti-insulin antibody levels. Diabetes Res (1991) 16:181–4. 1802485

[177] Elias D Bone AJ Baird JD Cooke A Cohen IR. Insulin-mimicking anti-idiotypic antibodies in development of spontaneous autoimmune diabetes in BB/E rats. Diabetes (1990) 39:1467–71. doi: 10.2337/diab.39.12.1467 2245874

[178] Oak S Gilliam LK Landin-Olsson M Törn C Kockum I Pennington CR. The lack of anti-idiotypic antibodies, not the presence of the corresponding autoantibodies to glutamate decarboxylase, defines type 1 diabetes. Proc Natl Acad Sci USA (2008) 105:5471–6. doi: 10.1073/pnas.0800578105 PMC2291139 18367670

[179] Tanasa RI Trad A Lange H Grötzinger J Lemke H. Allergen IgE-isotype-specific suppression by maternally derived monoclonal anti-IgG-idiotype. Allergy (2010) 65:16–23. doi: 10.1111/j.1398-9995.2009.02104.x 19624555

[180] Okamoto Y Tsutsumi H Kumar NS Ogra PL. Effect of breast feeding on the development of anti-idiotype antibody response to f glycoprotein of respiratory syncytial virus in infant mice after post-partum maternal immunization. J Immunol (1989) 142:2507–12. doi: 10.4049/jimmunol.142.7.2507 2926142

[181] Accolla RS Gearhart PJ Sigal NH Cancro MP Klinman NR. Idiotype-specific neonatal suppression of phosphorylcholine-responsive b cells. Eur J Immunol (1977) 7:876–81. doi: 10.1002/eji.1830071211 75802

[182] Weiler IJ Weiler E Sprenger R Cosenza H. Idiotype suppression by maternal influence. Eur J Immunol (1977) 7:591–7. doi: 10.1002/eji.1830070903 411678

[183] Ryelandt M De Wit D Baz A Vansanten G Denis O Huetz F. The perinatal presence of antigen (p-azophenylarsonate) or anti-mu antibodies lead to the loss of the recurrent idiotype (CRIA) in A/J mice. Int Immunol (1995) 7:645–52. doi: 10.1093/intimm/7.4.645 7547692

[184] Martinez C Toribio ML de la Hera A Cazenave PA Coutinho A. Maternal transmission of idiotypic network interactions selecting available T cell repertoires. Eur J Immunol (1986) 16:1445–7. doi: 10.1002/eji.1830161121 2430812

